# Multispecialty Enterprise Imaging Workgroup Consensus on Interactive Multimedia Reporting Current State and Road to the Future: HIMSS-SIIM Collaborative White Paper

**DOI:** 10.1007/s10278-021-00450-5

**Published:** 2021-06-15

**Authors:** Christopher J. Roth, David A. Clunie, David J. Vining, Seth J. Berkowitz, Alejandro Berlin, Jean-Pierre Bissonnette, Shawn D. Clark, Toby C. Cornish, Monief Eid, Cree M. Gaskin, Alexander K. Goel, Genevieve C. Jacobs, David Kwan, Damien M. Luviano, Morgan P. McBee, Kelly Miller, Abdul Moiz Hafiz, Ceferino Obcemea, Anil V. Parwani, Veronica Rotemberg, Elliot L. Silver, Erik S. Storm, James E. Tcheng, Karen S. Thullner, Les R. Folio

**Affiliations:** 1 Department of Radiology, Duke University, Durham, NC USA; 2 PixelMed Publishing, LLC, Bangor, PA USA; 3 Department of Abdominal Imaging, MD Anderson Cancer Center, Houston, TX USA; 4 Department of Radiology, Beth Israel Deaconess Medical Center, Boston, MA USA; 5 Radiation Medicine Program, Princess Margaret Cancer Centre - University Health Network, Department of Radiation Oncology, University of Toronto, Toronto, ON Canada; 6 Departments of Radiation Oncology and Medical Biophysics, University of Toronto, Toronto, ON Canada; 7 University of Miami Hospitals and Clinics, Miami, FL USA; 8 Department of Pathology, University of Colorado School of Medicine, Aurora, CO USA; 9 eHealth & Digital Transformation Agency, Ministry of Health, Riyadh, Saudi Arabia; 10 Department of Radiology and Medical Imaging, University of Virginia, Charlottesville, VA USA; 11 PuraJuniper, Toronto, ON Canada; 12 Norton Healthcare, Louisville, KY USA; 13 Health Technology and Information Management, Ontario Health (Cancer Care Ontario), Toronto, ON Canada; 14 Department of Surgery, Virginia Tech Carilion School of Medicine, Roanoke, VA USA; 15 Department of Radiology and Radiological Science, Medical University of South Carolina, Charleston, SC USA; 16 Memorial Health System, Springfield, IL USA; 17 Division of Cardiology, Southern Illinois University School of Medicine, Springfield, IL USA; 18 Radiation Research Program, National Cancer Institute, Bethesda, MD USA; 19 Department of Pathology, The Ohio State University, Columbus, OH USA; 20 Dermatology Service, Memorial Sloan Kettering Cancer Center, New York, NY USA; 21 Argentix Informatics, Vancouver, BC Canada; 22 Department of Radiology and Medical Education, Salem VA Medical Center, Salem, VA USA; 23 Department of Medicine, Division of Cardiology, Duke University, Durham, NC USA; 24 PenRad Technologies, Inc, Buffalo, MN USA; 25 Lead CT Radiologist, NIH Clinical Center, Bethesda, MD USA

**Keywords:** Enterprise imaging, Multimedia, Reporting, Interoperability, HIT standards

## Abstract

Diagnostic and evidential static image, video clip, and sound multimedia are captured during routine clinical care in cardiology, dermatology, ophthalmology, pathology, physiatry, radiation oncology, radiology, endoscopic procedural specialties, and other medical disciplines. Providers typically describe the multimedia findings in contemporaneous electronic health record clinical notes or associate a textual interpretative report. Visual communication aids commonly used to connect, synthesize, and supplement multimedia and descriptive text outside medicine remain technically challenging to integrate into patient care. Such beneficial interactive elements may include hyperlinks between text, multimedia elements, alphanumeric and geometric annotations, tables, graphs, timelines, diagrams, anatomic maps, and hyperlinks to external educational references that patients or provider consumers may find valuable. This HIMSS-SIIM Enterprise Imaging Community workgroup white paper outlines the current and desired clinical future state of interactive multimedia reporting (IMR). The workgroup adopted a consensus definition of IMR as “interactive medical documentation that combines clinical images, videos, sound, imaging metadata, and/or image annotations with text, typographic emphases, tables, graphs, event timelines, anatomic maps, hyperlinks, and/or educational resources to optimize communication between medical professionals, and between medical professionals and their patients.” This white paper also serves as a precursor for future efforts toward solving technical issues impeding routine interactive multimedia report creation and ingestion into electronic health records.

## Introduction

A city description enriched with an annotated map of streets and neighborhoods, embedded images and videos of landmarks, tables and graphs of citizenry demographics, and corresponding hyperlinks to additional content provides a better understanding of the city than would a textual description alone. To-scale timelines of a city’s past events effectively convey the city’s history to readers. Multimedia integration with textual elements has been shown to increase the quality and quantity of knowledge transferred because different forms of information are processed and integrated by a consumer’s brain [1–3]. Information consumers shift their attention between visual and textual elements integrated together in a single location or user interface depending on their needs at that moment. Organizations with experience studying captivating educational presentations recommend the integration of multimedia with descriptive tables, graphs, captions, and annotations in order to create engaging content [4, 5]. Similar visual communication principles guide content creation in the advertising and entertainment industries [6, 7].

In the fields of healthcare education and knowledge sharing, reputable medical research journals encourage manuscript writers to include illuminating graphics, tables, and online videos to effectively and efficiently convey results and conclusions to readers [8, 9]. Similarly, providing hands-on 3-D printed tactile anatomic models of patient abnormalities improves understanding of disease processes and ultimately surgical care over cases with exclusively textual descriptions of abnormalities [10–12]. As an interactive online map integrates information sources to help a consumer comprehend and use complex city information, clinical interactive multimedia reporting integrates several forms of textual and imagery data to help physicians comprehend a patient’s condition. Unfortunately, most clinical still image, video, and sound creation today is accompanied by only a separate exclusively textual description, rather than with interactive multimedia and text integration.

In August 2019, a workgroup of volunteers from the Healthcare Information and Management Systems Society (HIMSS) and the Society for Imaging Informatics in Medicine (SIIM), the HIMSS-SIIM Enterprise Imaging Community, convened the interactive multimedia reporting (IMR) workgroup to address this technical and clinical opportunity. The IMR workgroup’s purpose was “to serve as a multidisciplinary forum to share interactive multimedia reporting successes and challenges, and to spur innovation in across imaging-centric medical imaging subspecialties.” In doing so, the workgroup adopted a consensus definition of IMR as “interactive medical documentation that combines clinical images, videos, sound, imaging metadata, and/or image annotations with text, typographic emphases, tables, graphs, event timelines, anatomic maps, hyperlinks, and/or educational resources to optimize communication between medical professionals, and between medical professionals and their patients.”

Medical still images, video, sound, and modality-generated multimedia may be created from [13] the following:

- Diagnostic imaging or imaging-informed therapies, such as ultrasonography, radiography, fluoroscopy, CT, MRI, and nuclear medicine.
- Evidentiary documentation modalities in imaging-centric specialties, such as visible light dermatoscopes, endoscopes, microscopes, and ophthalmologic cameras.

Textual elements and communication aids which may supplement and enrich the above multimedia may include the following:

- Structured and synoptic reporting having structured headers consistently used for a specific scenario.
- Structured data element requirements and values (responses), such as tumor measurements. Structured outlines and data elements permit standardized collection, transmission, storage, retrieval, and sharing of data between clinical and research information systems and entities [14–16].
- Typographical emphases, including bold, underline, italicized, multicolor, and highlighted fonts, as well as varied fonts.
- High yield, representative key images, or video timestamps that clearly depict relevant findings, perhaps annotated with arrow, ellipse, text, or similar salient labels.
- Tables and graphs summarizing physiologic measurements (e.g., echocardiography ejection fraction) or lesion size (e.g., CT tumor volume) tracked over time.
- Graphical timelines organizing transactional, encounter-based care events into a to-scale, longitudinal patient health or organ-specific graphic user interface [17–20].
- Anatomic schematic diagrams identifying the physical location of a finding, such as for localizing a skin lesion or a coronary artery stenosis. Schematics can add clarity if two lesions being tracked occupy one anatomic location or if that anatomic location is difficult to describe in words.
- Hyperlinks to additional information or functionality. Hyperlinks in a report viewed on a patient portal may launch patient-level educational material. For healthcare professionals, links in a report may link to reference articles. Hyperlinks may launch synchronous communication tools such as chat or video teleconferencing between report consumer and creator, or asynchronous tools such as secure email with patient and study context. Hyperlinks in a graphical timeline have been used to launch medical viewers directly to a referenced finding or launch relevant clinical documentation such as radiation oncology therapy or surgical notes [21].

### The Structured Reporting Continuum

There is a variety of dictated, templated, standardized, form-based, checklist, structured and synoptic reporting across healthcare. Most clinical documents and reports are created by dictation, with or without the use of speech recognition software, and with or without the use of scenario-based templates to increase productivity and uniformity. Structured data entry into a scenario-specific application or form (contextual reporting) is possible, though usually only applied to highly stylized, high-volume, repeatable tasks. The recurrent use of consistent organization in reports about a particular subject is variously referred to as “structured” or “synoptic” reporting depending on the specialty and locale [22, 23]. In some cases, the layout, the required content, and the vocabulary used may also be standardized to various degrees. The transmitted result may be encoded in some structured format with preservation of machine-readable structured data elements and codes or remain textual, with the structured organization and data elements not recoverable without natural language processing (NLP). The scope of standardization may be constrained to one individual, group, practice, health care system, geographic region, or entire specialty. Standard requirements may also serve to improve quality, in the manner of a checklist.

Formal reconciliation of the definitions of these terms is outside of this whitepaper scope. For the purposes of this paper, “structured reporting” encompasses use of a pre-defined organization, with or without the specification of individual data elements (concepts and value sets, questions with predefined answer choices), with or without formal standardization of the layout, content, and representation. This is sufficient to describe the utility of structured reports compared to those that are unstructured, in the context of producing interactive multimedia reports.

In the subsequent specialty-specific sections, we describe the current state and long-term goals of interactive multimedia reporting in several imaging-centric medical specialties.

## Radiology

Shortly after Roentgen’s November 8, 1895, discovery of X-rays and their medical applications, imaging findings, and clinical consultative impressions were conveyed via handwritten summaries on paper prescription forms [24]. The evolution of these original handwritten reports progressed to human transcribed and/or typewritten prose textual summaries. In the late 1990s and early 2000s, the voice-recognition prose of today grew more common [25–27]. Radiology reporting increasingly includes structured elements based on standard lexicons in addition to the traditional prose reporting style, enabling research, interoperability, and better communication [28–31]. Radiology interactive multimedia reports and their precursors have long been described but remain underutilized [32–38].

### Current State

Plain text prose reporting remains the norm for the majority of radiology exams. Reports usually include consistent templated section formatting. Data elements from other systems, such as the electronic health record (EHR) Reason for Exam, are defaulted into the report. Radiology report data, even data created as structured in the reporting application, are commonly passed into the EHR unstructured.

Diagnostic radiologists most commonly create reports to answer diagnostic questions posed by those clinical providers primarily responsible for a patient’s care. Thus, radiology’s early IMR development aims to meet other providers’ needs, for example, a group of medical, surgical, and radiation oncologists interested in findings of a diagnostic radiology CT. IMR for oncologic purposes may be a competitive differentiator for practices, as they may integrate a number of features that make consuming images and text together more user friendly for groups of downstream physicians [39–44].

Structured and synoptic data lend themselves far better than prose to generating interactive multimedia reports due to being able to automatically associate data element, images, and downstream actions. Breast imaging and CT lung cancer screening governing bodies have mandated structured lexicons with evidence-based, expert-derived structured elements [45, 46]. Discrete lesion measurements may translate into interactive multimedia report lung cancer CT volume calculations, graphs, and tables. In the setting of anatomic segmentation machine learning, measurement annotations may automatically populate anatomic schematics depicting pulmonary location(s). Increases in lesion size above a threshold percentage may trigger automated referrer messaging, report typographical emphases, and populate values to a patient disease timeline. Numerical values for series and image number may drive image thumbnail passage into the report. Unfortunately, with even radiology structured reporting still relatively uncommon, something as simple as the passage of structured measurements from viewers into defined fields is not a typically employed workflow.

Ideally, more radiology reports would incorporate the passage of discrete, distinguishable, quantitative, and annotation data with minimal physician intervention. Radiologists are participating in efforts to standardize the concepts and data elements used in reporting, including use of Logical Observation Identifiers Names and Codes (LOINC) and Systematized Nomenclature of Medicine Clinical Terms (SNOMED CT) ontology expansion, Radiological Society of North America (RSNA) RadLex®, and RSNA-American College of Radiology (ACR) Common Data Element (CDE) development. ACR and RSNA have created standardized reporting templates integrating these structured terms [47–54].

**Fig. 1:**
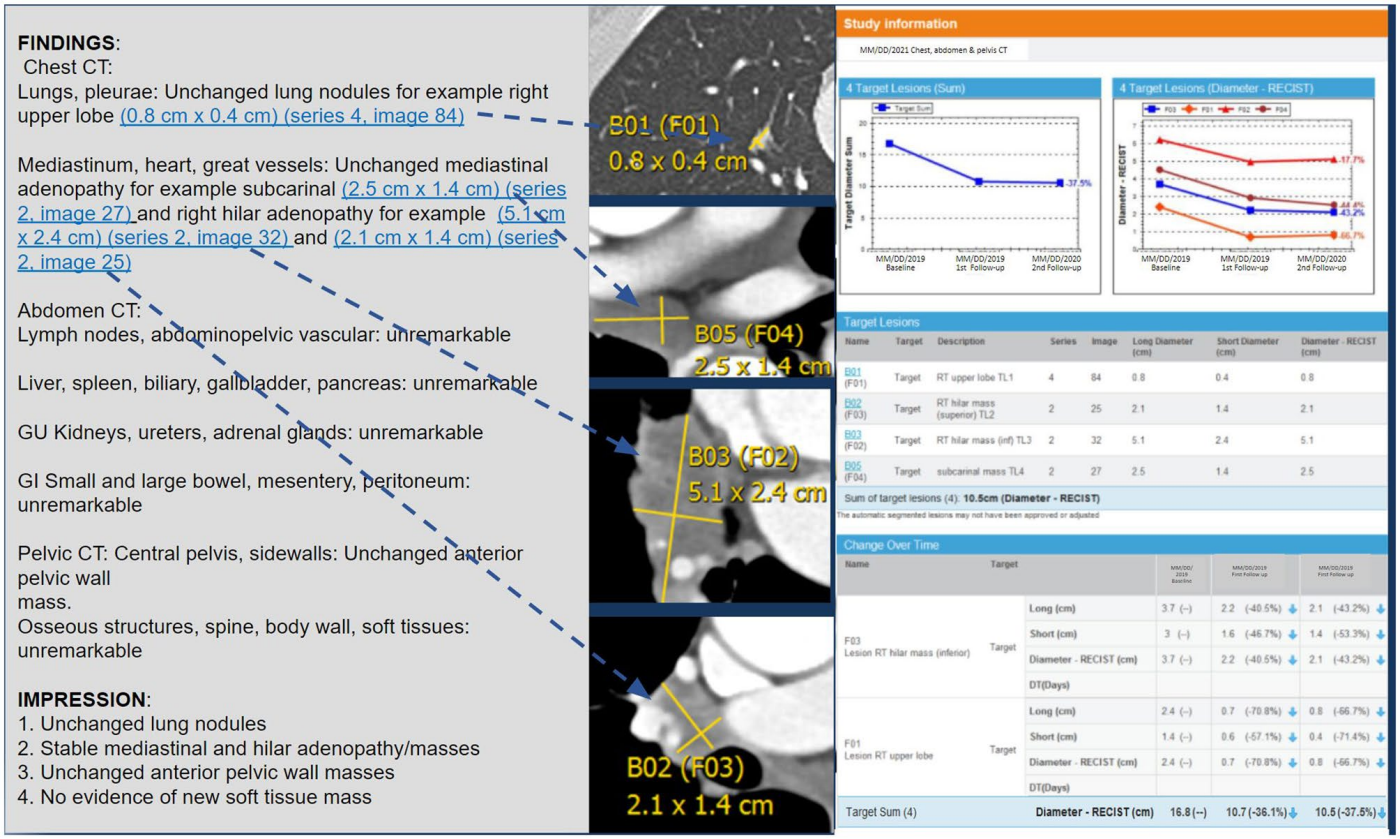
Current state radiology interactive multimedia report from a single vendor integrated diagnostic viewer and report creation system. Note hyperlinks (blue and underlined) from text to referenced images, lesion level tables, graphs, and annotation measurements

Most PACS systems used by radiology departments for diagnostic reporting allow radiologists to annotate images and tag “key” images the interpreting radiologists feel are especially noteworthy. Interpretation software often allow image thumbnails to be included in dictated text, yielding simple multimedia reports. More advanced reporting solutions enable report text hyperlinks, or hypertext, to launch a full functionality image viewer to the hyperlinked finding on-demand. Direct launch from a report to relevant images with measurements and other annotations eases image and text consumption, as reviewers need not launch and log into a separate viewer, scroll through lower yield images, or exert time and effort to visualize the findings of concern (Fig. 1). Such linking to the most salient referenced imaging findings would save referrer time and improve service to the user.

In complex patients with multiple lesions all perhaps having undergone different interventional, systemic, and surgical therapies at different times, keeping track of individual lesion histories is challenging with an unstructured and verbose report Findings paragraph. Longitudinal tracking tables and graphs may synthesize need-to-know lesion level information for report consumers, such as bi- or tri-dimensional measurements or volumes, region of interest calculations of beam attenuation, or physiologic measurements of tissue perfusion. Such data may come from radiologists manually creating structured measurements or calculations in a diagnostic viewer, or from technologists performing similar tasks at the modality level, the latter particularly in ultrasound. With tables and graphs at the individual finding or calculation level, new lesions generate new tables and graphs, while lesions being followed over time can undergo tracking using calculations such as the Response Evaluation Criteria in Solid Tumors (RECIST) [55]. Additional relevant information like tumor marker tracking can be added along these lesion timelines.

When interoperability permits such integrations, potential transcription errors are minimized, and radiologist speed increases as structured measurements are shared from the viewer to the created report [56, 57]. Consensus standards such as Digital Imaging and Communications in Medicine (DICOM) Grayscale Presentation State (GSPS), DICOM Structured Reporting (SR), and SR template TID1500, specifically, provide industry agreed-upon mechanisms to communicate, store, locate, and manage such annotations [58, 59]. The annotations and resultant structured data can serve as expert labeling on routine reporting for machine learning algorithm validation and development and have been described in an IHE Radiology Integration profile for AI Results [60, 61]. Hyperlinks in reports can also launch email, screen sharing, or paging or chat functionality for communications between radiologists and referring providers. Some sites have tested “patient friendly,” educational report descriptions of significant findings launched out of hyperlinks [62]. Hyperlinks also can launch quality improvement feedback mechanisms for downstream physicians [63–65].

### Road to the Future

**Fig. 2:**
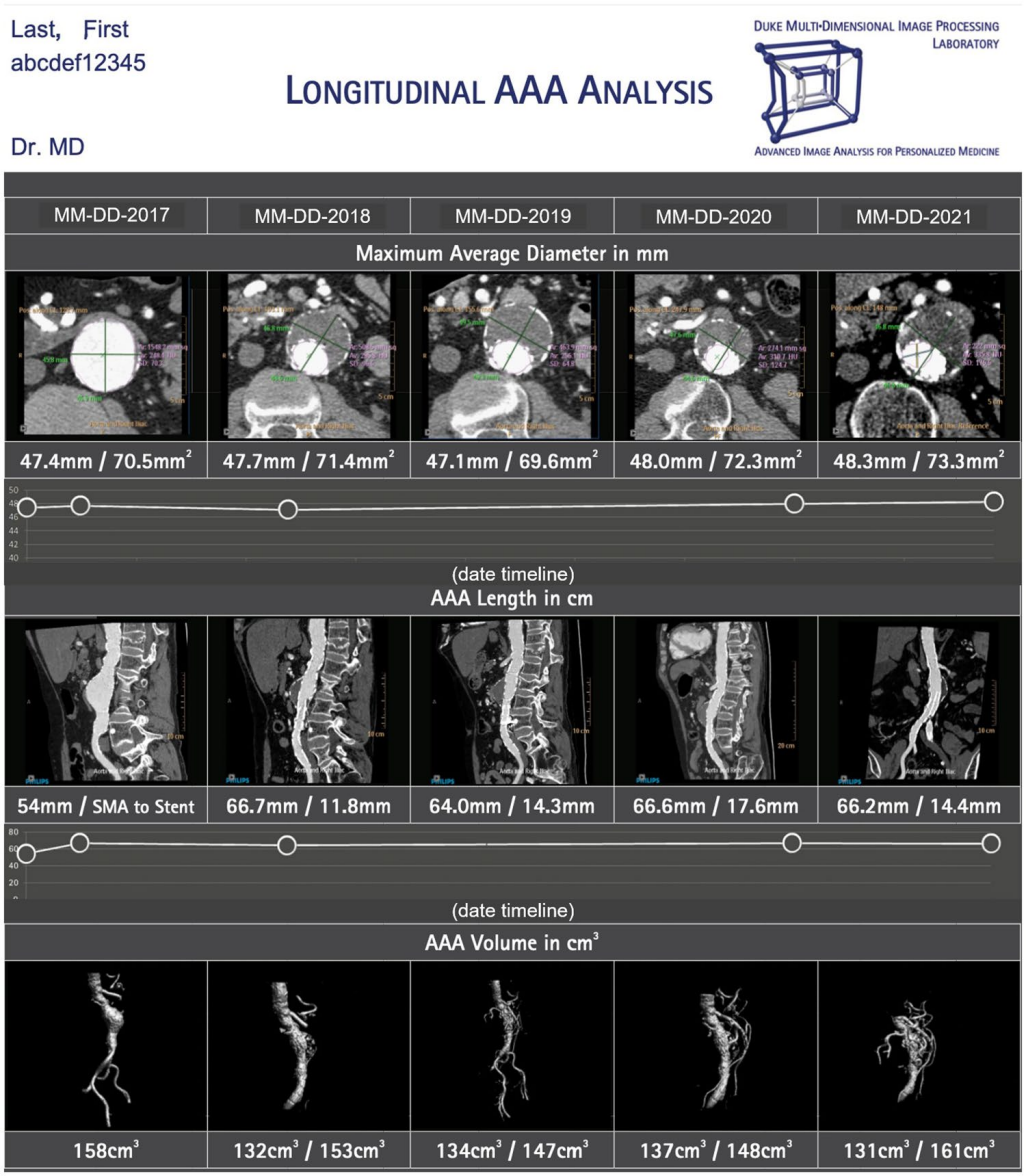
Current state radiology interactive multimedia PDF report including axial CTA (computed tomography angiogram) images, multi-planar reformats (MPR), and volume renderings (VR) serially tracking an aortic aneurysm patient with subsequent stent and endoleak across timepoints. This single-page document permits review of multiple imaging encounters at once, moving left to right. Such interactive multimedia reports at one author’s institution (CJR) require manual creation upon request, in this case for surgical planning and patient education. The VR and centerline MPR rotational datasets in this PDF document can be engaged and manipulated by mouse click-and-drag interactivity

Improved diagnostics and therapeutics have translated into longer patient survival and in turn a greater need to curate long-term care-related information throughout the patient journey. Combining data currently captured on transactional or encounter bases into a long-term timeline of patient health and organ-specific disease is warranted (Fig. 2). Radiologists could either generate and own these timeline-based interactive multimedia reports themselves or act as contributors to content curated and owned by other specialties, perhaps oncologists. Radiologists would increase their own professional value in doing so. IMR remains a rarity in radiology despite the benefits of integrating longitudinal images, text reports, hyperlinks, tables, timelines, annotations, and other tools. The most common barriers to radiology IMR include dominant system interoperability, the breadth of radiology imaging, limited utilization of structured reporting, and overcoming real or perceived disincentives to create interactive multimedia reports.

#### Dominant System Interoperability

The primary challenge remains interoperability gaps between radiology’s most commonly used diagnostic image viewers, speech recognition-based reporting systems, and EHRs. The growth of HL7 FHIRcast has started to bridge this gap, albeit slowly and still with only a minority of vended applications. Some radiology practices utilize innovative combined viewer and report creation applications, which lower the IMR creation barrier significantly, though this is only available at a minority of sites [66]. Even if radiologists could easily create and send out such reports, proprietary and site-specific output interfaces and integrations entering the downstream EHR often degrade interactive multimedia report quality and value by displaying the interactive multimedia report in a portable document format (PDF) reader with limited functionality. Today even simple typographical emphases like bold, underline, and italics cannot be reliably transferred from reporting system to EHR. Report hyperlink functionality may raise security challenges when access is expected outside of the host medical facility.

If these barriers across vendor systems cannot be overcome, an alternative could be radiologists directly creating interactive multimedia reports within the EHR itself. Unfortunately, many EHRs currently have limited capabilities to incorporate images efficiently. Optimal EHR natural language parsing automation to create such reports remains elusive. Finally, many EHRs have not deployed requisite capabilities to display interactive graphs or tables of measurements. These may be development opportunities given EHR roles in tracking longer-term patient outcomes.

#### Breadth of Radiology Imaging

Radiologists utilize a wide variety of modalities and procedures to diagnose and treat a wide range of patient presentations and diseases. The clinical justifications and technical requirements for IMR vary by modality and individual case findings. For example, in most radiology studies on patients with no abnormalities, structured format, typographic emphases, and added educational hyperlinks are potentially valuable, but annotations and graphs may not be in many cases. In reports from uncomplicated interventional procedures such as central line placements, clinical hyperlinks may be valuable, but tables, and structured physiologic or anatomic data passage may be less so. In addition, in creating interactive multimedia report tables and graphs, a radiologist will recognize that longitudinally imaged lesions may be imaged in different planes, during different contrast conditions, or on different modalities; these differences may lead to measurement variations and potentially erroneous conclusions regarding lesion progression or stability [67]. In hyperlinking report text to images, a radiologist may need to choose a single best key image depiction from several available series, images, or secondary captures; this single image may similarly misrepresent lesion progression or improvement to the consumer if that single image looks changed in size, such as between two studies with differing fields-of-view. These common clinical variations underscore the requirements for flexibility, user direction, and standard procedure recommendations in interactive multimedia report creation across the breadth of radiology studies.

#### Structured Reporting

High-quality radiology templated and structured reports offer precise conclusions and management recommendations to peer physicians: support administrative activities, such as those for dashboarding or registry participation, and ensure completeness for revenue capture by requiring clinical elements and offering optional sections for additional information. Unfortunately, radiologists have typically dictated free text prose for decades. To create and consistently utilize structured reporting takes an initial push from champion radiologists, payers, societies, or hospitals as radiologists, like many physicians, perform clinical duties under significant time pressures. Thus, it is essential that new advanced reporting options not be detrimental to report turnaround time. If new advanced reporting techniques are not quick and easy, they are unlikely to be adopted, even if available. It has been shown that radiologists do adopt interactive multimedia reporting techniques in clinical practice if the technology is deployed in a satisfactory manner [68].

Cross-society and industry discussions are ongoing to standardize and map body parts across ontologies so that “underlying” imaging anatomy, such as the bladder, may be related to external “surface” anatomy such as the pelvis or genitalia. These cross-ontology efforts would assist with linking radiology images to those from other specialties, for example, from post-traumatic emergency room bladder ultrasound and urographic CT to cystoscopic and external infraumbilical visible light skin photography. This cross-ontology effort would support multispecialty structured report interoperability and facilitate easy tagging of sensitive or privacy-protected skin photography in archives [69].

#### Overcoming Disincentives

For abnormal radiology studies, referrer providers may risk not visually reviewing images or multimedia elements if a given longitudinally imaged lesion is reported as stable compared to priors; the report of stability itself is presumed to be “enough” information to appropriately manage that patient. Of course, in patients with stable lesions, structured data measurement passage for interactive multimedia report timelines, graphs, and tables is valuable if that lesion later grows. Nevertheless, if there is significant manual work to create interactive multimedia reports, radiologists may be tempted to ask if the curation effort for a given stable patient was “worth it.”

Even if system interoperability permitted easily created interactive multimedia reports, radiology practices commonly interpret studies from numerous provider clinics and hospitals. Systems receiving the radiology report from the imaging practice may only be able to accept a plain text report. It is not realistic to expect individual radiologists to triage the desire to create interactive multimedia reports for certain practices with EHRs or receiving systems capable of accepting the output, but not other practices without such systems. Thus, there remains a clear need to enhance the versatility and compatibility of the end-user systems frequently used to display reports and to find practical solutions to the security and authentication issues that may interfere with interactive functions.

## Cardiology

Virtually all cardiac imaging can be thought of as radiology extensions dedicated to the visualization of cardiovascular structures. As with radiology, traditional reporting in cardiology has been prose, with the verbose narrative of procedures and findings remaining the predominant procedure reporting format. Nonetheless, structured reporting is slowly replacing traditional verbose reporting, including the incorporation of IMR concepts to enhance communication and more efficiently convey content [70]. Like radiology, the transformation in cardiology brings parallel opportunities and challenges as those described for radiology above.

An additional driver of structured reporting in cardiology has been the need for “good data” about clinical processes, care delivery, and patient outcomes for quality assessment and performance improvement purposes. Many of these improvements in care delivery have specifically been enabled by disease and device registries that require the submission of well-formed, high quality, semantically consistent information as discrete data [71]. Integral to the function of registries are data dictionaries that define the clinical concepts to be captured; beyond registries, multiple professional society initiatives have further extended the defined cardiovascular lexicon [72, 73]. Given the tens of thousands of clinical concepts just in cardiovascular medicine necessary for structured reporting, the natural need is for workflow tightly integrated with dataflow such that data captured at the point of care by the entire healthcare team is used to generate clinical documentation, for registry data submission, and for other secondary analyses. This structured reporting approach reduces redundancy and clinician burden while allowing all members of the healthcare team to practice at the full extent of their education, training, and experience.

### Current State

DICOM Structured Reporting templates are widely used to communicate quantitative data between a number of cardiovascular modalities and the respective reporting systems [74, 75]. A prime example is in echocardiography and vascular ultrasound, where multiple dozens of measurements are routinely conducted (out of a menu of several hundred possible measurements) and uploaded to reporting systems. Automated data interchange avoids the need for manual copy/paste or dictation operations inside report building. Elimination of accompanying transcription errors makes report generation more accurate and time-efficient. However, these implementations are typically unique to each installation, with the capability to annotate and utilize (limited) multimedia extensions restricted to proprietary software available only at the cardiologist’s diagnostic workstation. Unfortunately, final reports sent to the EHR system are typically PDF files, stripped of dynamic annotations, hyperlinks, and other IMR elements. Even when available, the limited IMR capabilities are leveraged only at larger medical centers, with most institutions still manually copying this otherwise digital information through transcription or re-dictation into final procedure reports. Furthermore, approaches outside of the modalities of echocardiography and electrocardiography are inconsistent and not standardized.

**Fig. 3:**
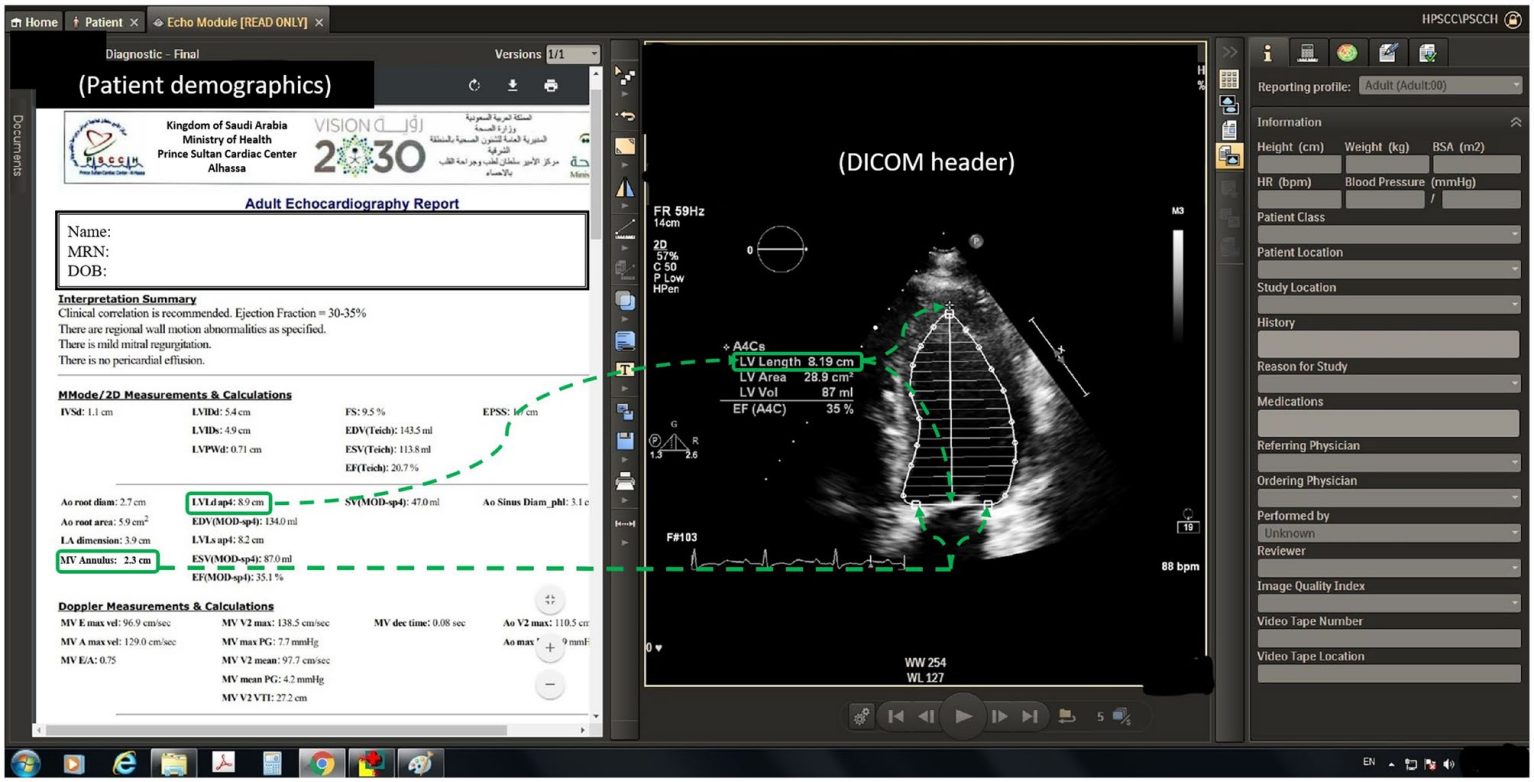
Mock-up echocardiogram interactive multimedia report incorporating multimedia elements and structured text. When the user clicks on report structured text measurements in a future interactive report, the hyperlink could open the relevant cine frames and annotation, in this case the left ventricular length or mitral valve annulus diameter (dotted arrows)

Reflecting the IMR concept, a limited number of laboratories have advanced to the point of formally embracing structured reporting that includes multimedia elements. These reporting systems typically facilitate the direct transfer of measurements from the modality to the reporting system database along with the capture of qualitative and semi-quantitative assessments and interpretations of key imaging findings as data (e.g., segmental left ventricular wall motion, degree of valvular regurgitation or stenosis on echocardiography). As illustrated in Fig. 3, graphical representations of the assessments and interpretations are represented both textually and diagrammatically. Critically, the underlying database structure is designed to capture the data once (whether as a structured choice or via an interactive diagram) while showing the information in the two separate and semantically identical representations (text and graphics). Optionally, since a typical echocardiography study can capture nearly 100 sets of still and video images, illustrative static images can be imported into the report, or more importantly, key findings can be associated with hyperlinks that directly open the image sequence that best illustrates the finding.

Similarly, a limited number of cardiac catheterization laboratories use software reporting solutions that facilitate the incorporation of multimedia elements and potentially interactive hypertext into the report. Compared with the limited anatomic variability of the structure of the heart (and thus the appropriateness of standardized diagrams that universally represent segments of the heart), coronary artery anatomy varies considerably. Whereas an echocardiography study features a series of reproducible quantitative measurements, cardiac catheterization is typically almost entirely qualitative, particularly the characterization of the angiographic findings (e.g., degree of calcification, lesion length, presence of thrombus) as well as the assessment of the percent stenosis of coronary artery lesions.

**Fig. 4:**
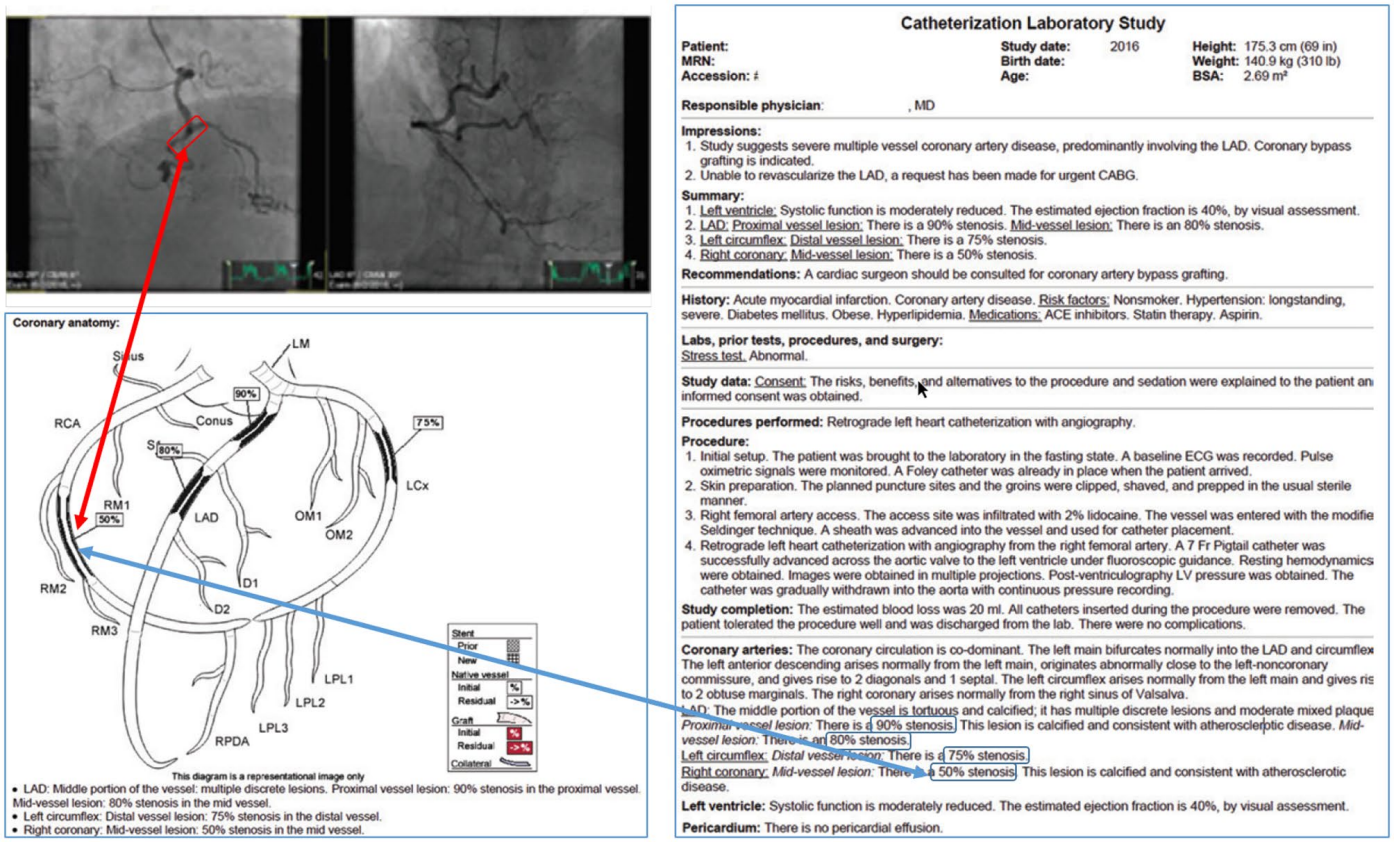
Mock up cardiac catheterization interactive multimedia report incorporating cine loops of interest, typographical emphases, standardized formatting, and anatomic schematic rendered coronary stenotic lengths and percentages interactive with hyperlinked text. Cine images would be associated with the appropriate schematic location (red arrow) and hyperlinked structured text (blue arrow). This interactivity would be present for all described lesions

Although text can be used to describe a lesion (e.g., “long diffuse 50% stenosis of the mid right coronary”), a more effective and arguably accurate method for the consumer of the report is to draw the lesion on a generic coronary artery diagram while including the key image which illustrates the lesion (Fig. 4). Of note, the information is not precisely semantically interoperable—the semi-quantitative representation of lesion length on the diagram relative to other branches simply cannot be accurately described with text. Separate data stores are needed for the diagrammatic and the text-based information in this situation. Also, interactive relationships between hypertext descriptions can be connected to the imaging findings and graphical representation to complete the description most appropriately (arrows in Fig. 4).

### Road to the Future

While the IMR opportunity is already being realized in a limited number of institutions, impediments to the widespread cardiovascular implementation of multimedia reporting are numerous. Many of the limitations relevant to radiologists also apply to cardiologists. There must be dedicated innovation and development in cardiovascular information systems, modalities, and EHRs in the context of an uber framework of vendor neutrality, the end result being an enablement of IMR within the EHR irrespective of the system used for report generation. These EHR IMRs may be leveraged to increase patient engagement and understanding of their disease processes to facilitate management and treatment such as medication and exercise regimen compliance. Finally, it must be simple for physicians to generate and populate structured patient data in IMR, easier than dictating a verbose report. This may be accomplished by leveraging participation of procedural technicians to capture information as data to be used in procedure report generation as well as incentivizing health care systems to support this.

## Pathology

Anatomic pathology, which includes both histopathology and cytopathology, is, by its very nature, a highly visual discipline with many opportunities for integrating image-based multimedia into reports. Opportunities exist in clinical pathology as well, including in the microbiology, hematology, flow cytometry, and cytogenetics labs. In many respects, pathology was an early adopter of multimedia reporting as laboratory information systems have long been able to incorporate images into reports. Interactive multimedia, however, has generally been absent from pathology reports, but the recent introduction of digital pathology into routine pathology workflows will increase opportunities to integrate multimedia into pathology reports.

### Current State

To the outside observer, it would appear that the anatomic pathology report has changed little in the last hundred years. While handwritten reports gave way, in turn, to the typewriter, the word processor, and the laboratory information system (LIS), the report itself is still predominantly composed of narrative text [76, 77]. Notable exceptions include Bethesda System-related reporting elements in cytopathology reports and “synoptic” (structured) cancer reports, which in this case are often (but not always) built on discrete data present in the LIS [78]. Most LISs are also capable of embedding images, tables, graphs, and other forms of static media in reports. Unfortunately, for those pathology practices with an LIS that is interfaced to (as opposed to integrated with) an EHR, the report is usually rendered as plain text, removing all discrete data and embedded multimedia.

#### Multimedia in Pathology Reports

As noted above, multimedia is not new in pathology reporting, and “best of breed” LISs have been able to embed images in reports since the mid-1990s. The embedded images are almost always photomicrographs illustrating key diagnostic findings. In surgical pathology, these are typically brightfield images, but some subspecialty areas (such as renal pathology and dermatopathology) also include transmission electron micrographs and fluorescence photomicrographs. Macroscopic (“gross”) images, which are commonly acquired on surgical resection specimens, play an important role in internal documentation, but are rarely embedded in reports. Exceptions are beginning to emerge concerning this last point. For example, the American College of Surgeons (ACS) National Accreditation Program for Rectal Cancer requires that images of rectal resections are available to the clinical treatment team [79]. The most straightforward way to accomplish this is to embed these visible light images in the pathology report.

Although images and other multimedia can be embedded in pathology reports, the version presented to clinicians may not include the embedded content. Pathology practices deliver reports to clients using a variety of means. For some practices, this still includes “hard copy” reports that are printed, faxed, or emailed directly from the LIS, and these reports will retain the original formatting and any embedded multimedia. More commonly, clinicians access reports in a downstream EHR or Health Information Exchange (HIE) that receives them from the LIS via an electronic results interface. These interfaces are usually implemented in plain text (typically HL7 v2), and images, tables, and graphics may be stripped in the process. In other cases, the EHR will accept a base64-encoded PDF version of the report but may still display the plain text version by default. In contrast, an LIS that is integrated as a module in a larger EHR product should preserve the original formatting and display any embedded images and multimedia.

#### Synoptic (Structured) Reporting

Since 1986, the College of American Pathologists (CAP) has published cancer protocols that serve as "checklists" designed to ensure completeness, standardization, and consistency in cancer reporting [80]. The protocols reflect the current American Joint Committee on Cancer (AJCC) staging and World Health Organization (WHO) histologic classification. Today, 100 electronic Cancer Checklists (eCCs) from 94 protocols cover a wide range of cancers while an additional 9 templates standardize cancer biomarker reporting [81].

The CAP synoptic cancer protocols include a standardized case summary format that defines a set of required core data elements, conditional data elements, and optional data elements. These elements include information such as tumor site, tumor size, histologic type, margin status, lymph node status, tumor (T), node (N), and metastasis (M) staging, and other significant diagnostic and prognostic findings. Unlike in other imaging specialties, synoptic reporting in pathology is the norm, as use of the CAP cancer protocols is required for laboratory accreditation by CMS-deemed accreditation programs (e.g., the CAP and The Joint Commission) and for accreditation by the American College of Surgeons (ACS). Essentially all pathology labs in the USA and Canada use synoptic reporting for primary cancer resections.

It should be noted that while all pathology synoptic reports have a structured appearance, they may or may not contain underlying discrete data elements that can be extracted without NLP. Cancer reporting in pathology can be classified into six levels from pure narrative text (level 1) to a structured synoptic format utilizing a standardized reporting language, discrete storage of data elements, and a standardized encoding such as SNOMED CT (level 6) [82]. While the CAP and ACS mandate the use of a synoptic format and certain data elements (level 3), they do not require discrete data elements or mapping to a standard encoding. In contrast, Cancer Care Ontario (CCO) has mandated the use of level 6 synoptic cancer reporting in the Canadian province of Ontario [83].

#### Image Acquisition

Digital imaging was introduced in pathology in the 1980s and has grown to encompass a range of modalities [84]. Conventional digital photomicrographs are produced in several ways, including transmission electron microscopy (TEM), fluorescence microscopy, brightfield microscopy, and polarized light microscopy. Some pathology labs have even adopted digital x-ray radiography for the evaluation of specimens [85]. Whole slide imaging (WSI), invented in the late 1990s, has gained a clinical foothold in a few centers, both in Europe and more recently in the USA, with the FDA’s clearance of several digital pathology systems [86].

Whole slide imaging creates a high-resolution image from a glass slide by using a digital slide scanner [87]. Many labs scan some portion of their slides for clinical diagnostic use or immunohistochemistry. It is believed that only a handful of pathology labs in the world currently have a 100% digital workflow, i.e., digitize all their slides and then perform clinical diagnosis digitally rather than using a microscope. Those sites that use WSI for clinical purposes interface their digital pathology system to their LIS, permitting digital slides to be opened in a case-oriented context.

#### Image Storage and Retrieval

Though DICOM defines a standard for pathology image storage, including WSI, it is not yet widely adopted for this purpose, perhaps reflecting the lack of widespread use of digital pathology and WSI in the first place. Vendor-specific proprietary formats often based on TIFF are widely used in research. Options for storage include file system-based storage, generic image management systems, instrument-specific image management systems, LIS-based storage, pathology-specific PACS systems, digital slide repositories for WSIs, and DICOM-compliant PACS and VNAs. With the exceptions of fluorescence images and WSI, pathology images are relatively small (less than 20 megapixels) and can be represented as RGB images using common image formats. While fluorescence images can be rendered to RGB, preservation and manipulation of individual channels are preferable and require software designed for this purpose. WSI storage and retrieval are even more specialized due to the size of the image (multi-gigapixel), the unique way it is structured, and the need to dynamically interact with the image.

#### DICOM for Pathology

DICOM Working Group 26 (WG-26) is charged with supporting and developing DICOM’s use in the Pathology Domain and has made considerable progress in supporting pathology images, including WSIs [88, 89]. WG-26 has coordinated with numerous vendors to demonstrate interoperability in a series of Digital Pathology Connectathons in the USA and Europe [90]. While DICOM promises a standards-driven future in pathology imaging, its clinical use in pathology is still limited, and as yet no FDA-cleared digital pathology systems for sale in the US actually employ it natively [91].

#### Image Annotations

Image annotations are commonly used to perform measurements, mark key features, highlight regions of interest, and visualize image analysis results. In pathology, these annotations include both bitmapped overlays (e.g., heatmaps) and vector-based shapes (e.g., polygons and points). Unfortunately, the annotations used in pathology are even less well standardized than the images themselves. Each vendor has created its own method of annotating images. Although many of these mechanisms are superficially similar, they differ considerably in their implementation. Interoperability between these software packages is virtually non-existent, which has led most vendors to simply “burn” annotations into an image’s pixel data before transferring it to a foreign storage system. Needless to say, such annotations are no longer machine-readable and hold little promise for interactive multimedia.

The DICOM standard provides for image annotations, in the form of DICOM Structured Reports (which preserve the semantics of the annotation) and Presentation States (which record the text and graphics but lose semantics), and DICOM Segmentations that encode annotations pixel-by-pixel. Specific extensions to these standard mechanisms have been defined to allow them to work with tiled pyramidal multi-frame WSIs. The DICOM WG-26 WSI Annotations Ad-hoc Group has recently been formed to explore such issues [92]. The group is specifically exploring the use of DICOM SR TID 1500 to encode small numbers of human-generated annotations as contours and categorical and quantitative results. It is also developing a new extension to DICOM to support compact representation of vast numbers of similar annotations, such as might be created by AI processing of a high-resolution WSI (e.g., millions of nuclei). The DICOM annotation approach is likely the best answer to this issue in pathology, but it does, of course, require the images themselves to be stored as DICOM objects. As noted above, the adoption of DICOM in pathology has been minimal to date.

### Road to the Future

Multimedia interactive reporting represents the evolution of reporting in pathology. The emergence of DICOM for pathology and the migration of digital pathology into clinical settings are essential elements that should enable its adoption. At present, truly interactive multimedia reporting exists neither within the LIS nor in downstream systems. Implementing multimedia reporting within the laboratory (i.e., within the LIS) is a goal that might be achievable in the near term and would allow prototyping and deploying interactive multimedia reports under the auspices of a single vendor without immediately introducing the complication of multivendor support. Successful implementation of interactive multimedia reporting within the LIS could then be extended to downstream systems, including EHRs.

**Fig. 5:**
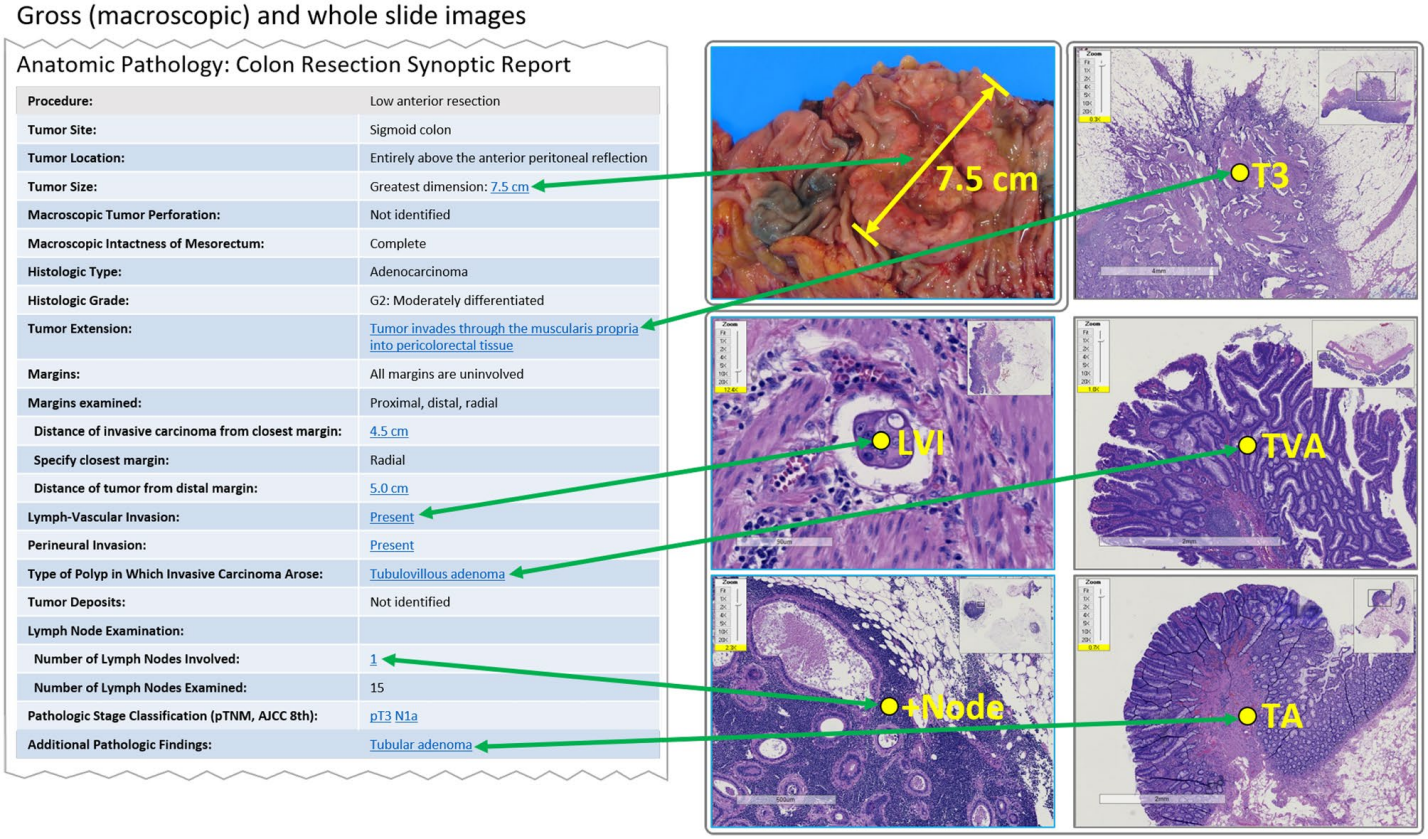
Mock up pathology interactive multimedia synoptic report. This synoptic report is derived from the CAP’s “Protocol for the Examination of Resection Specimens From Patients With Primary Carcinoma of the Colon and Rectum” v. 4.1.0.0. Elements in the synoptic report could be enhanced with hyperlinks to annotated gross and histologic images opened in a DICOM viewer. Annotations on the images could include dimensional measurements, arrows or polygons to draw attention to particular areas, or operational specifics such as slide or block number

Figure 5 outlines a vision of how interactive multimedia reporting might be implemented with regard to synoptic cancer reporting. While this is the most obvious and highest yield report section that could be enhanced in this way, it is not the only section amenable to the incorporation of interactive multimedia. Other standard report sections that would benefit from interactivity include the final diagnosis, diagnostic comment, microscopic description, and gross description. Even if these sections remain free text, interactive hyperlinks could be embedded in the text with linkage, for example, to specific microscopic features, diagnostic terms, or macroscopic findings.

While pathology has a long history of multimedia reporting, progressing to the next level of interactive multimedia reporting will be challenging. Several pieces are in place for interactive multimedia reporting, including extensive use of digital imaging. Other parts are emerging, such as interoperability standards for imaging and workflow (DICOM and IHE PaLM [Integrating the Healthcare Enterprise’s Pathology and Laboratory Medicine domain]) and complete digitization of workflows via WSI and digital pathology [93]. Unfortunately, the adoption of standards like DICOM is coming slowly to pathology. This issue is exacerbated by the large number of image acquisition and storage vendors in the pathology market and the significant investment that labs have already made in hardware and software with poor interoperability. These issues are especially prevalent in the macroscopic (gross) image and digital photomicrography segments of the pathology market, where discussions of using standards for retrieval and storage of images and annotations are practically non-existent. Pathologists must become more discriminating customers and demand the adoption of interoperability standards for all digital imaging going forward to modify the attitudes of these vendors.

Another technical challenge to interactive multimedia reporting is the lack of sophisticated information exchange between the LIS and EHR (and other downstream systems). No standard mechanism exists currently that allows EHRs to receive pathology reports that embed or link to interactive multimedia. Interfaced pathology reports are typically sent as a plain text report, sometimes accompanied by a PDF version. EHR-integrated LISs fare somewhat better as the reports are native. Embedding links in PDF reports is one solution to implementing simple interactive media, but security restrictions in the EHR may stymie this approach. Of course, this problem is not specific to pathology and affects all specialties that report out of interfaced information systems.

Implementation of interactive reporting is further complicated by the many settings in which pathology labs operate. While hospital-owned labs and academic labs may be intimately associated with their respective hospital or health system, private labs can have highly complicated relationships with multiple clients and may send pathology reports to many and various downstream systems. These complicated business relationships often manifest as multiple layers of IT security that must be navigated if images, annotations, or other interactive content is to be accessed or retrieved across network firewalls or domains. Clearly, these concerns need to be considered if IMR is to be implemented broadly in pathology.

Finally, pathologists themselves present a significant challenge to implementing interactive multimedia. Today even something as simple as embedding a static photomicrograph in a report can be controversial. Even though all modern LISs are capable of doing this, very few pathologists do so, and many departments actively discourage it. Pathologists are reluctant to do this for several reasons. The most commonly cited reasons are that it is time-consuming, it does not add value to the report, and it raises potential legal issues [94]. Since resistance to the adoption of multimedia already exists, it is important that future implementations of interactive multimedia directly address these concerns.

## Endoscopy

Multiple IMR opportunities exist in visible light laparoscopy, arthroscopy, bronchoscopy, colonoscopy, endoscopic ultrasound, and other types of endoscopic imaging during the examination and treatment of maladies in various body cavities. For example, during knee arthroscopy, evidentiary images are commonly captured of normal or abnormal menisci, articular cartilage, cruciate ligaments, and any structures after surgical repairs have been performed. Capturing still images of a red mass behind the tympanic membrane or video of vocal fold mobility during phonation are increasingly common practice in otolaryngology. DICOM standards and IHE Endoscopy Domain profiles currently support these applications, but as in other disciplines, forward progress is slow and often heterogeneous [95–97].

### Current State

IMR adoption in endoscopy remains fragmented across clinical subspecialties. Endoscopic and surgical suites capture a combination of endoscopic non-DICOM visible light images, as well as DICOM fluoroscopic, endoscopic ultrasound, and transcutaneous ultrasound images and video during a procedure. That data may be stored using a vendor’s proprietary endoscopic equipment into the same vendor’s reporting system or an additional reporting system. Multimedia reports combining free-text dictation and/or structured reporting plus illustrative images are exported as PDF documents for storage in an EHR. Optimally these multimedia data elements (i.e., images and video) would be individually tagged with metadata and made accessible in the EHR, but that does not exist in EHR systems today for the same technical and security reasons cited elsewhere as IMR hurdles.

**Fig. 6:**
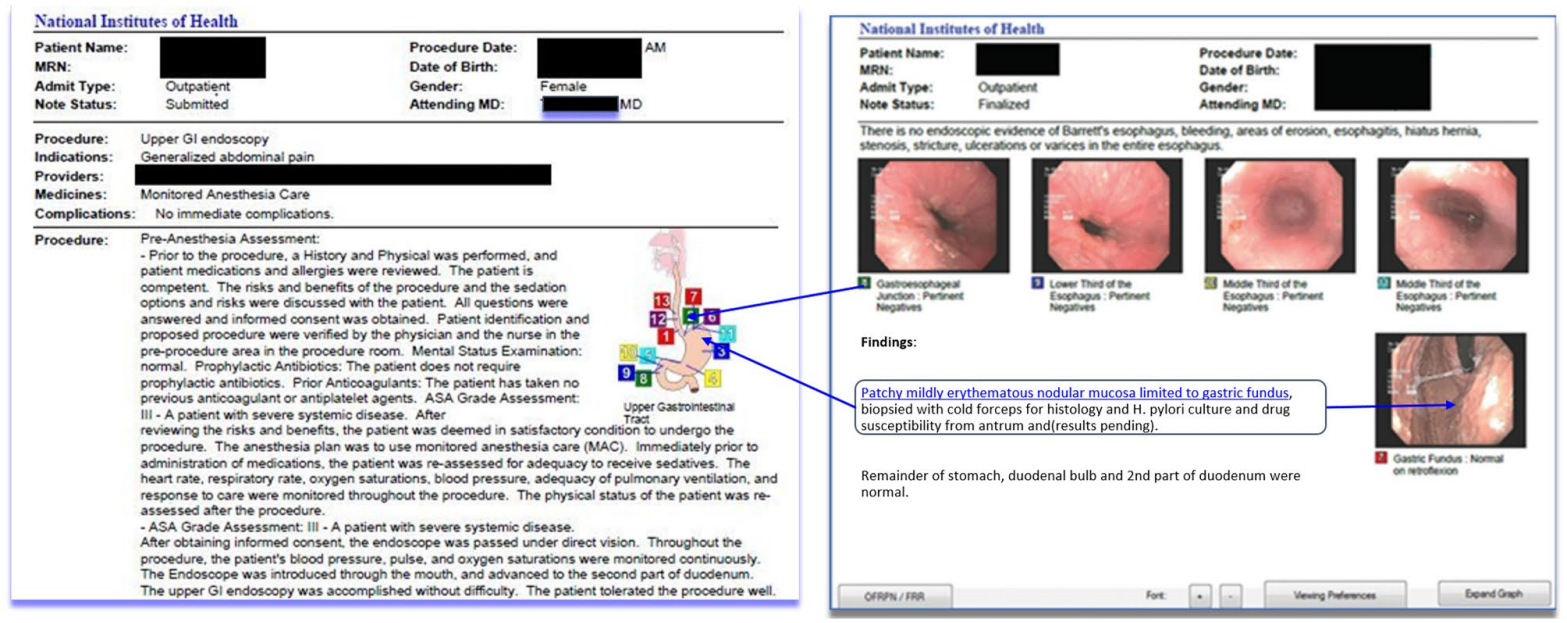
Mockup upper endoscopy interactive multimedia report available in the EHR including anatomic schematic graphic with numerical relations to report descriptions (*courtesy of Les Folio, DO*)

Gastroenterology has been leading the endoscopic specialties with the development of quality standards for procedural documentation incorporating elements of IMR [98]. Key images from an upper GI endoscopy combined with a graphic of the stomach might demonstrate pertinent regions along the greater curvature of the stomach where ulcers are found or demonstrate abnormalities such as bleeding or erythema (Fig. 6). After capturing images during a procedure, gastroenterologists can annotate the key images and assign them to the stomach graphic using a third-party reporting application separate from the EHR. These key images and video clips may or may not be available beyond the PDF report output in a vendor neutral archive. Text descriptions of relevant GI findings in a structured format may be available for assignment using drop-down menus, and those prepopulated descriptions may be assigned to the schematic lesion automatically or via a mouse click on the graphic.

**Fig. 7:**
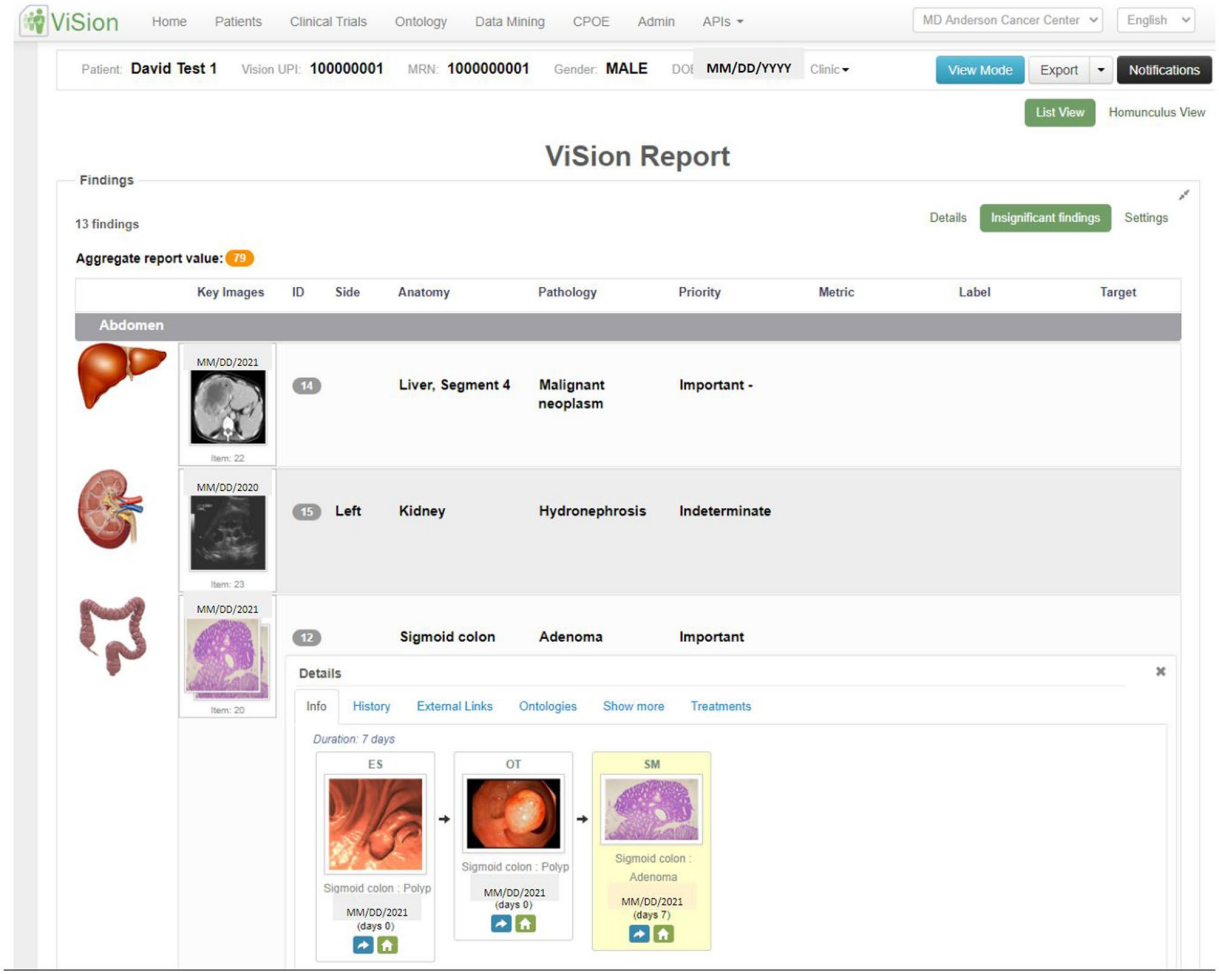
An integrated multimedia interactive report including anatomical findings from different modalities (CT, US, endoscopy) and clinical disciplines (radiology, pathology, gastroenterology). The bottom finding presents a colonic polyp timeline with CTC, conventional colonoscopy, and histology images from the same finding (*courtesy of David J. Vining, MD*)

**Fig. 8:**
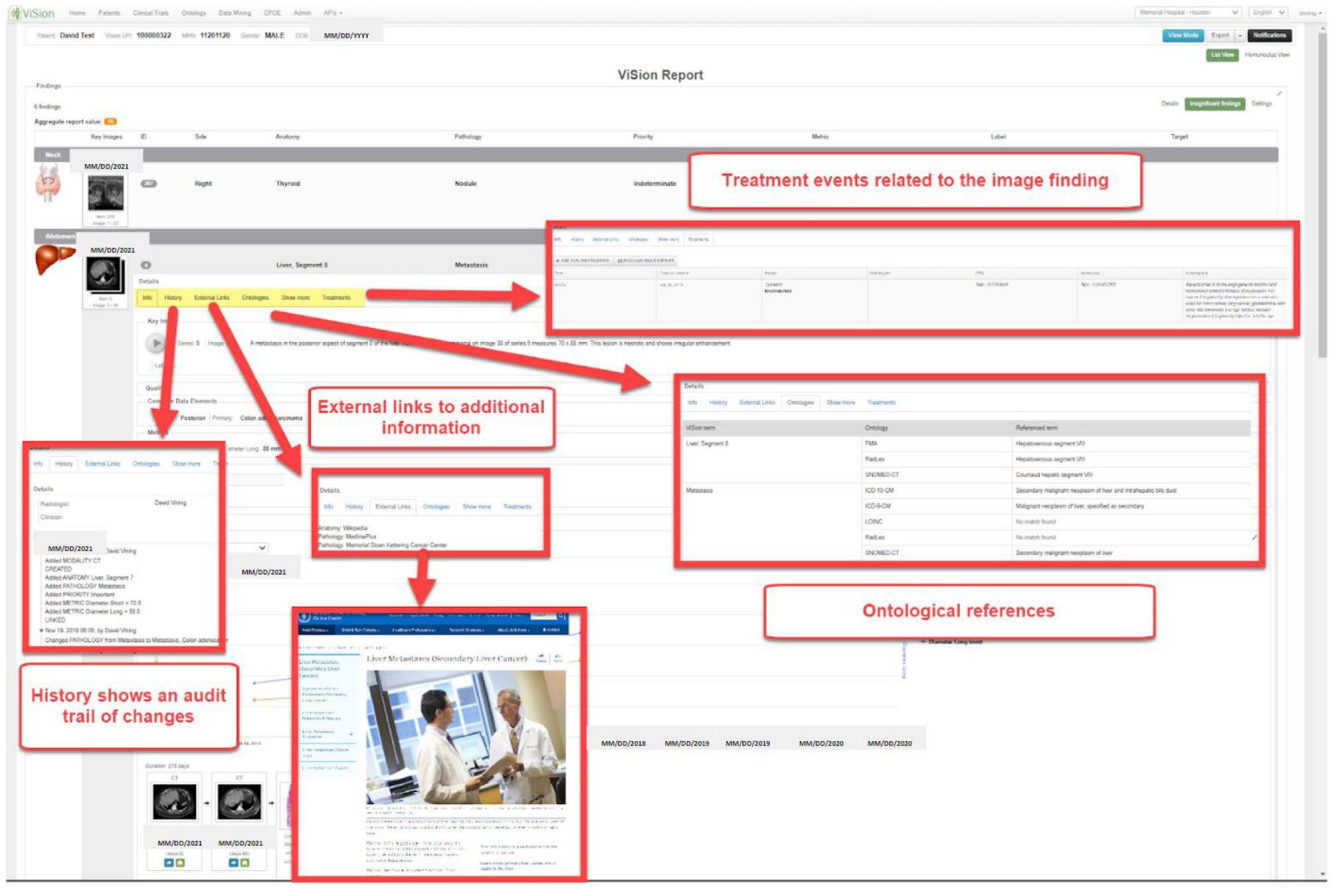
An expanded view of details from one of the report findings in Fig. 7 demonstrating interactivity with access to an audit trail of report changes, external links to related information for clinician and patient education, treatment events affecting the image finding, and cross-references to standard ontologies including ICD-10-CM, LOINC, RadLex, and SNOMED CT (*courtesy of David J. Vining, MD*)

Correlation of conventional colonoscopy and CTC (a radiological exam reconstructing CT or MR images in a 3D virtual environment) findings is important when CTC follows an incomplete colonoscopy, or when colonoscopy is recommended for the evaluation of screening CTC findings. This correlation is important since conventional colonoscopy has been shown to not always correlate with preoperative localization of colorectal cancer due to the “telescoping” effect of colonoscopy or due to the absence of clearly defined anatomic landmarks in some cases [99]. Interactive multimedia reports for CTC findings have existed since the 1990s. Formalized structured reporting standards were introduced in 2005 [100, 101]. More recently, investigators are integrating multimedia CTC findings with endoscopy and pathology in timelines to illustrate the evolution of disease (Fig. 7). These timelines can also incorporate related treatment events (e.g., surgery, radiation, drugs) and lab data (e.g., tumor markers) to present a holistic view of the patient. In the CTC report, images and video from multiple encounters and specialties are incorporated into a single composite report. Structured data within this report may include measurements and analyses made by gastroenterologists and other medical specialists. In addition, this type of report has hyperlinks to other types of information, including external references, audit trail of report changes and status updates, and ontological cross-references (Fig. 8). This type of report is rich in IMR elements that are not accessible after transmission to the EHR. The same shortcomings that limit the incorporation of non-DICOM visible light and DICOM images into the EHR and sharing of data across institutions remain a hurdle to endoscopic specialties.

### Road to the Future

Among endoscopy specialties, gastroenterology was the earliest adopter of basic IMR by incorporating still images of endoscopic findings at the point of care. The need to document clinical findings during an active examination at the point of discovery helped drive early development. In the future, GI procedure interactive multimedia reports could incorporate radiology DICOM images/video from motility studies, upper GI radiological exams, contrast enemas, small bowel follow-throughs, endoscopic retrograde cholangiopancreatography (ERCP), or CTC. With the adoption of vendor neutral archives and digital video recordings, it may be possible to archive entire procedures, which is infrequently done today. IMR could provide bookmarks into video streams to enable rapid access to specific video frames without a user having to watch or scroll through an entire video sequence, saving physician time and lowering the barrier to viewing the multimedia.

Endoscopists, and particularly gastroenterologists, may control multiple modalities (e.g., visible light, endoscopic ultrasound, fluoroscopy guidance) in the endoscopy suite that would be amenable to IMR in the future. Other endoscopy specialties employ fewer modalities (e.g., otolaryngology with ear, nasopharyngeal, or laryngeal exams) but similarly will benefit from more sophisticated interactive multimedia report generation, especially when combined with radiological imaging (e.g., temporal bone CT), surgical and/or pathological outcomes. An overarching aim of IMR is to increase the integration of endoscopic images and video into interactive multimedia reports that can assimilate multidisciplinary information to create a more patient-centric health record.

## Radiation Oncology

Therapeutic radiation doses are delivered in daily fractions through linear accelerators or brachytherapy remote afterloaders that derive input from treatment planning systems. Treatment planning systems import CT and MRI DICOM images for the radiation oncologist to segment (i.e., contour) anatomical structures. The radiation oncologist targets radiation at diseased tissue, such as the tumor or involved lymph nodes, while, if possible, avoiding healthy tissue to minimize treatment-related toxicities. Through a highly computational process, the treatment planning system simulates the placement and shaping of radiotherapy beams iterating variations on the consequent impact on radiation dose to structures of interest until an optimal dose distribution is obtained. The resulting treatment plan is then transferred to delivery devices. Controlling systems monitor the daily delivery of the radiation treatment as originally prescribed and planned.

### Current State

In 1987, the DICOM-RT extension was published, introducing five information object definitions (IOD) [102]. The RT Image object stores 2D images generated by treatment planning systems with color overlays to help visualize beams traversing the patient for comparison with verification images obtained immediately before treatment [103]. The RT Structure Set contains the actual contours drawn on CT, MR, US, or PET images to define targeted structures and organs-at-risk, and defines them as Regions of Interest (ROIs) [104]. Safety margins can also be included in the treatment plan to account for microscopic extension of the disease, geometric uncertainties due to organ motion, daily patient setup deviations. The RT Plan object contains all of the machine parameters required to deliver the optimized plan, the actual dose prescription, and the patient setup instructions [105]. In the case of a medical linear accelerator, the RT Plan describes the *control points* required to deliver the dose, each control point specifying gantry and collimator angles, the treatment couch positions, the dose actually delivered, and the description of the beam-shaping device used to collimate the radiation beam. The RT Dose object contains the radiation dose for every voxel across the image datasets for the optimized plan [106]. Finally, the RT Treatment Record contains a summary of the machine parameters recorded for each treatment session during delivery. DICOM-RT enables the transfer of images and treatment data between systems within and/or across institutions. The DICOM-RT extension has been used for decades to add RT-relevant structured information layers onto morphologic image datasets; this data can be used as foundational elements for radiation therapy IMR.

The practice of radiation oncology continues to evolve at a fast pace. Technical advancements such as daily 3D-image guidance and respiratory-gated CT imaging, and novel clinical applications such as the treatment of arrhythmias, have pushed for even more integration with other specialties and created demand for interoperability of various imaging modalities and reporting systems with DICOM-RT [107]. Other medical specialties, such as radiology, could exploit the RT Structure Set definitions and tools to clearly indicate tumor margins, size measurements, or sub-volumes of interest directly on the images to collaboratively guide therapy and develop quantitative imaging approaches. Similarly, one can envision that incorporating impedance imaging modalities or image-fusion of endoscopic images onto CT or MR images could increase the precision of target definition for radiation treatment. In parallel, advances in early cancer detection and treatment have translated into increased incidence and prevalence of patients living longer with their disease, in turn making the scenario of re-treatments more common. In these oncology patients, the capacity for seamless aggregation of longitudinal and semantically rich imaging and radiation treatment data is becoming a pressing need.

While some PACS vendors support the storage of DICOM-RT elements, not all incorporate visualization, creating some uncertainties for end-users and hesitation from radiation oncology departments to adopt an enterprise PACS for storage and viewing purposes. Hence, it is not surprising that, to date, the understanding and use of radiation oncology images and data remain limited to the confines of radiation oncology departments and specialists. This gap results in missed opportunities for collaboration and use of multi-dimensional data around patient care. For example, if a patient comes to the emergency department due to a radiation therapy-related toxicity, the emergency room physician could benefit from having a simplified record or interactive multimedia report of the radiation treatment location and dose delivered. Other care providers, radiologists especially, would find radiation treatment data valuable when reviewing progression of disease on follow-up imaging. Dentists could benefit from more granular spatial radiation dose information to bony and salivary structures to help them manage radiation toxicities or prevent the occurrence of complications to their interventions in the context of irradiated fields, such as osteonecrosis. Information regarding dose to particular organs such as skin can drastically change the management approach from topical treatments (i.e., radiation dermatitis) to investigation of disease persistence/recurrence with biopsy or excision (i.e., a suspicious lesion). Monitoring and management related to pacemakers and defibrillators may be influenced by their absorbed dose.

Therefore, a multimedia report with hypertext indicating irradiated volumes and the doses received by structures of interest will help various treating providers better manage the oncologic patient’s care. Another example of an activity where DICOM-RT-aware PACS and IMR would be desirable is the context of multidisciplinary tumor boards. By visualizing images with interactive multimedia reports simultaneously, radiation oncologists, medical oncologists, and surgical oncologists may converge quickly towards the most appropriate treatment course to recommend; sharing such data between departments of various medical specialties would also enable virtual consultations between clinicians and centers.

### Road to the Future

**Fig. 9:**
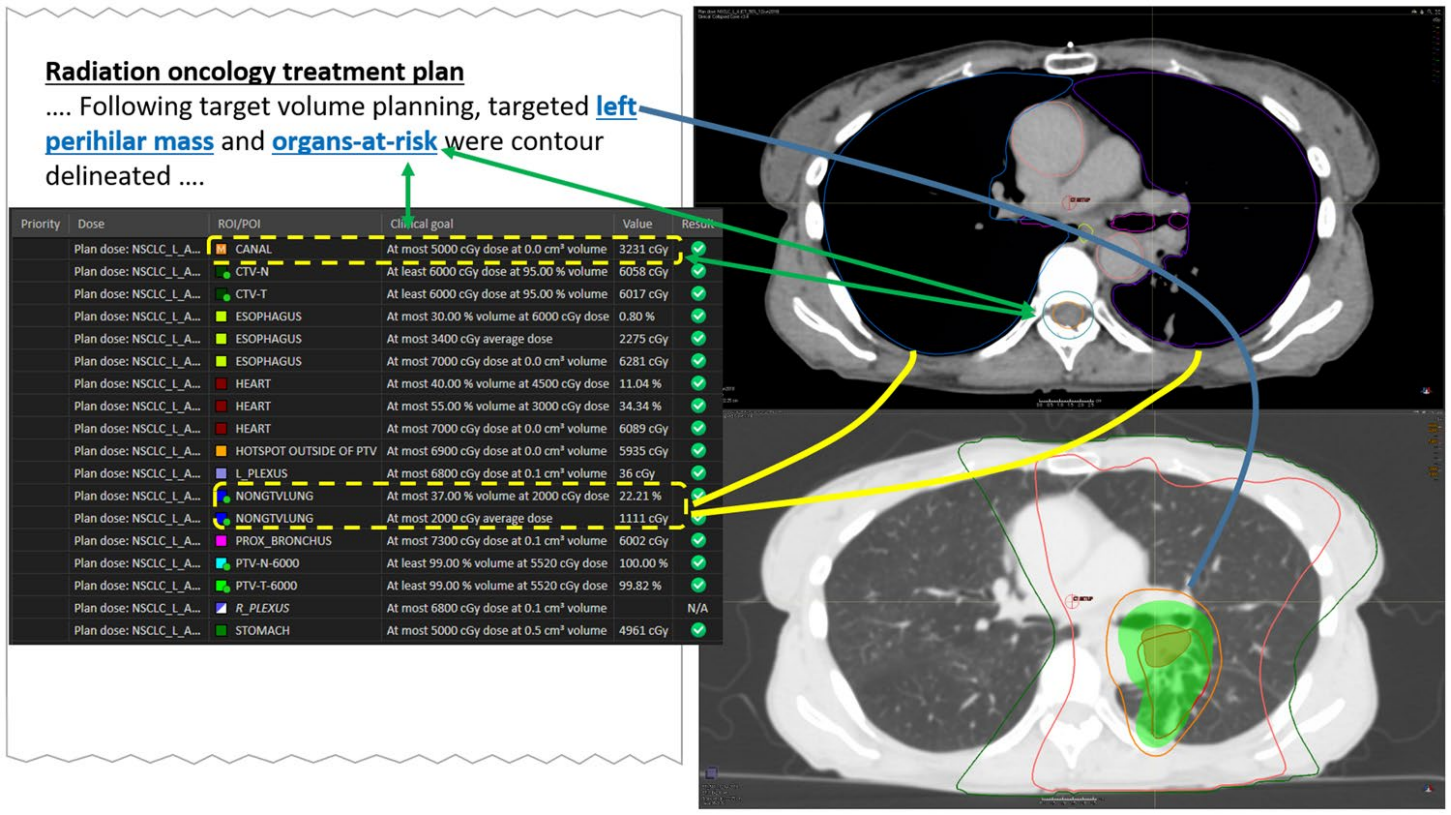
Mock up radiation therapy interactive multimedia planning map linking descriptions to lung window images depicting radiation concentration contour lines focused on a left perihilar mass (blue arrows). Itemized organ dose calculations with manual or automated anatomic annotations based on the plan may be saved as part of the interactive multimedia report to highlight possible sites of radiation induced complication, for example, to the nearby spinal cord (green arrows) or lung (yellow arrows)

Radiation oncology has a track-record with robust methods and existing specialized data standards (DICOM-RT) that allow the addition of information layers to image datasets. Hence, radiation oncology is uniquely poised to expand towards incorporating treatment planning and delivery images into a comprehensive and longitudinal patient care IMR including diagnosis, imaging, lab, and therapies curated by the primary oncologist, centering specialties around the oncologic patient’s care. Even a simple IMR (Fig. 9) incorporating a complex therapeutic planning map of delivered radiation plus a textual description would assist radiologists and other physicians caring for later complications or determining causes for progression. As the complexity and sophistication of radiation oncology methods and devices have increased, a new effort to define so-called Second Generation DICOM-RT objects is now well underway. Such new objects included more complex annotation capabilities and leverage the general purpose DICOM Segmentation mechanism. The IHE Radiation Oncology (IHE-RO) domain is developing new integration profiles based on the new mechanisms.

Oncologic treatments, including the management of their complications, often necessitate interdisciplinary approaches. Like radiology and pathology, radiation oncologists collaborate with many different clinical specialties. Radiation oncology IMR would need to be flexible to accommodate the various needs of this spectrum of consumers. Importantly, this could be an opportunity for those specialties to merge information with the oncologic and DICOM-RT data, such as treatment outcomes (i.e., survival, loco-regional control, distant-disease free survival) and graded toxicity reports, in turn informing the entire care team and enabling research to improve the therapeutic index and value of radiation treatment interventions. Radiation oncology IMR could then host and aggregate genuinely patient-centric care multimodal information emerging before, during, and after oncologic treatments. New understandings of disease control, toxicity outcomes, and (de)intensification strategies would hopefully come from this patient-centric approach. IMR should be considered as a unique opportunity for a collaboration framework between radiation oncology and collaborating disciplines.

## Dermatology

Dermatologic consultation may occur live in clinic, or electronically through real-time or asynchronous teledermatology. Imaging in dermatology is used as an adjunct to documentation to represent particular skin conditions at a given moment in time. Most dermatology images are acquired with off-the-shelf phones and tablets not using the DICOM standard. Less commonly, higher-resolution compact or digital single-lens reflex (DSLR) cameras are used, also without DICOM. Some clinics invest in 2D or 3D total body photography for patients with multiple suspicious or cancerous lesions to be tracked [108]. While 3D topology images are becoming more widespread and contain quantitative calibrated information, they typically require proprietary viewers with limited interoperability, and come with an increase in cost and complexity.

Dermatology patient privacy protection and privileged access are especially important considerations in total body photography, imagery of faces, graphic or sensitive body parts, and unique tattoos. The imaging data is ideally obtained for immediate transmission utilizing a secured wired or wireless network to an EHR, PACS, or vendor neutral archive (VNA) without local device storage. Manual transmission, such as via a portable memory card, to an intermediary system continues to be common but is not recommended due to security and patient-matching concerns.

Like most medical specialties that depend on reliable point of care visible light image acquisition, storage, and analysis, dermatology suffers from local and cross-institution workflow and dataflow variation. Workflows vary for review and annotation of acquired images depending on the clinical scenario, the archive and viewing platform, and location. Forums like the HIMSS-SIIM Enterprise Imaging Community build technical and workflow consensus and raise awareness. Encounter-based photos are typically annotated within an EHR office visit document (sometimes referencing photos or lesion IDs directly). Order-based workflows are usually cumbersome in dermatologist offices due to the frequency of captured photos obtained on-demand and without antecedent EHR orders placed [109].

Reporting of encounters may occur in the EHR, in a dedicated teledermatology platform, and/or in informal messaging such as email. Salient clinical photographs may or may not be pasted alongside the clinician report summary and management. Anatomic site labeling has historically been loosely based on SNOMED CT terminology to encourage consistent anatomical descriptions [110, 111]. Similar to other specialties, dermatology is establishing a specific lexicon that can encourage more structured reporting for many aspects of image annotation, including anatomic site, histopathologic diagnosis, and clinician impressions [112].

### Current State

Dermatologists review historical stored images most frequently to investigate for lesion progression, or during cross-disciplinary communication, such as with pathologists or wound care nurses and providers. Monitoring disease over time is particularly challenging in the setting of transactional, encounter-based documentation and very brief clinic visits sometimes having many clinical images per visit. Longitudinal tracking is especially challenging. As described above, there is also no specialty-wide, agreed-upon dermatology process for technical capture, transmission, or incorporation of photography into clinical documentation, much less longitudinal tracking [113]. There is also no operational consensus for specific lesion registration or calibration to allow quantitative measurement, with the exception of specialized modalities such as calibrated total body photography or optical coherence tomography. With the absence of these agreed-upon workflows and dataflows, IMR is distinctly uncommon in dermatology.

If efficiently integrated at the point of care, the DICOM standard would assist not just with archiving but the metadata capture necessary for interactive multimedia reporting. Additional efforts in the DICOM Dermatology WG-19 are addressing communicating the vast array of photography equipment, image types, and subjective photo preferences, including angles, lighting, and filters. Dermoscopy is the first dermatologic imaging modality to be addressed by the DICOM working group [114]. Future modalities to be considered include Reflectance Confocal Microscopy (RCM) and total body photography.

### Road to the Future

**Fig. 10:**
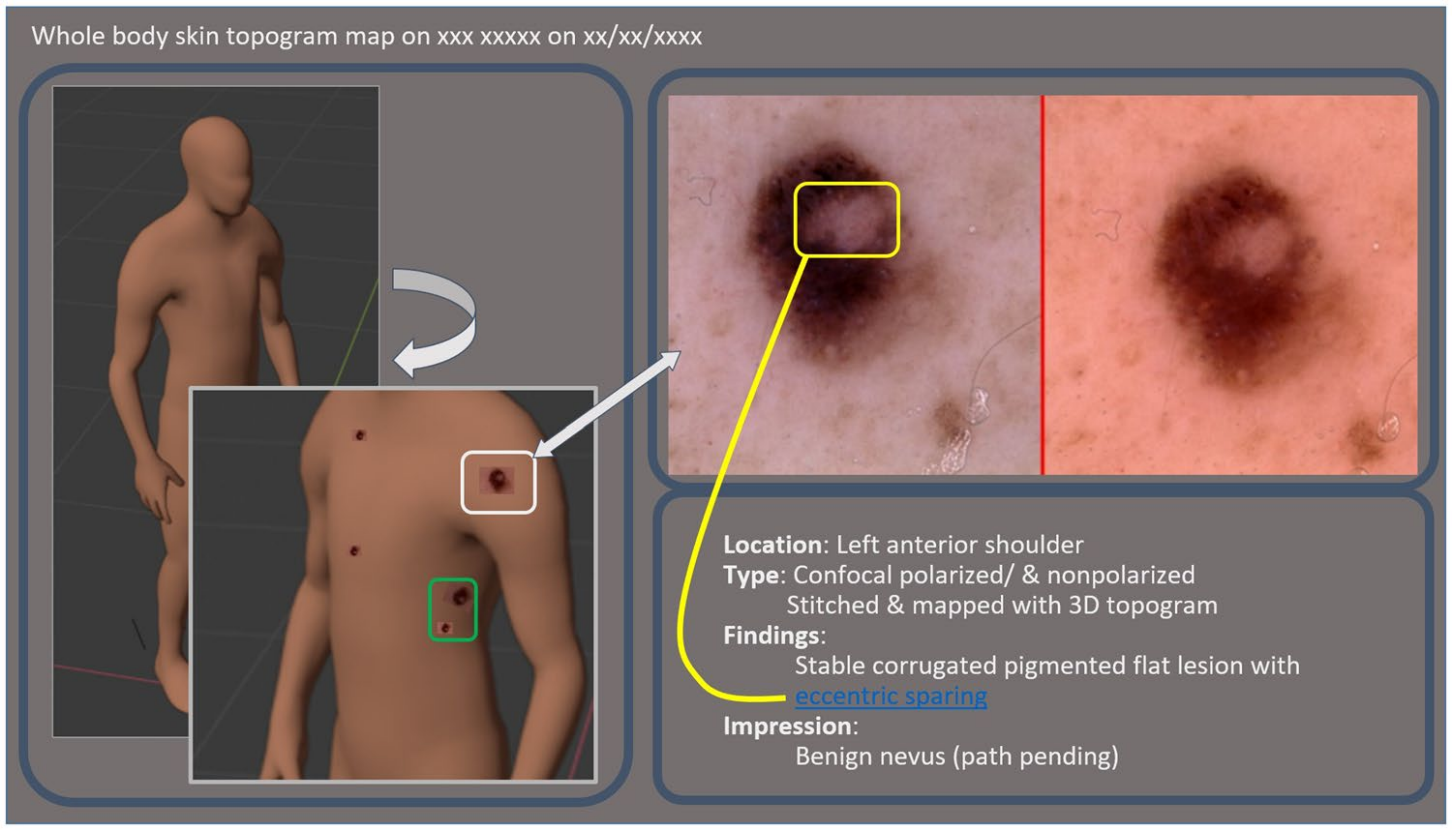
Mock up dermatology interactive multimedia report, parts of which are commercially available at this time, demonstrating non polarized and polarized light images of a lesion on the left shoulder integrated into 3D topography (white box and arrow). Dermatologists rotate the virtual patient, honing in on an area of interest, clicking on that area or saved key regions, which in the future would display annotated images associated to the hyperlinked textual descriptions (yellow arrow). The schematic body map is beneficial to clarify lesion location when there are multiple lesions sharing the same anatomic descriptor terms, here as shown on the left ribcage/flank (green box)

An ideal dermatology interactive multimedia report would include an interactive 3D topology schematic incorporating 2D high-resolution images, measurements, impressions, and pathology reports along a graphical timeline or filmstrip accessible from the EHR. The topology schematic would rotate and zoom to expose all external skin (Fig. 10). The topology would incorporate a standardized body part ontology where 2D images can be linked to cartoon lesion annotations, and associate report terms to the chosen lesion anatomic location. Explicit links, not dependent on descriptions or terminology, between lesion locations visible on 3D maps, whole body surveys and regional images to the corresponding closeup and dermatoscopic images are especially useful.

Structured document creation could be initiated by physician extenders or clinic staff, and ultimately edited and signed by dermatologists. The documentation would include information passed from an anatomic schematic, including all notated lesions, the number and modality of images of each lesion, and anatomic terms. EHRs would pass relevant information, such as history of present illness, medications, and past medical and surgical history. Dermatologists would include diagnostic impression, a structured management plan (e.g., “monitor,” “biopsy,” “excise”). Hyperlinks to the subsequent pathologic report and images for biopsied lesions would populate when those reports are complete. The structured data would be available for subsequent patient clinical follow-up tracking, and research, especially machine learning development. When complete, the interactive report would be accessible from the EHR.

In summary, IMR in dermatology is desirable but hampered by a number of factors. Highly variable clinical image capture hardware and workflows lead to limited adoption of storage and transmission standards that facilitate necessary metadata association. Anatomical description heterogeneity being the norm prevents tagging sensitive or identifiable images, and automated presentation of related images. Plus, EHR interactive multimedia report consumption, presentation, and reimbursement limitations referenced above in radiology and cardiology similarly apply to dermatology. Until these challenges are overcome, dermatology interactive multimedia report creation and sharing will remain limited [115–117].

## Ophthalmology

Ophthalmology, like pathology, endoscopy, and dermatology, relies heavily on visible light images. DSLR cameras capture external imaging, such as the eyelids and conjunctiva. Specialized digital retinal cameras capture color surface and angiographic retinal images. Slit-lamp micrography captures corneal, lens, and iris imaging. Ophthalmologic optical coherence tomography (OCT) is analogous to radiology ultrasound, but it uses light waves instead of sound. OCT reveals reflective differences within the layers of the retina, creating a 4-dimensional high-resolution cross-section of the retina and optic nerve.

To illustrate the importance of IMR using an ophthalmology use case, we will use glaucoma, a common eye disease, and one using several imaging modalities. Ophthalmologists evaluate the cup, contour, and color of the optic nerve head in diagnosing and following the course of glaucoma. Like other phenotypic body parts, the optic nerve head appearance varies throughout the healthy and glaucomatous populations. Tracking the optic nerve head over time helps determine if surgical intervention is warranted or if medical management is sufficient [118]. Timelines associated with IMR allow for visualizing and analyzing data from different diagnostic modalities while providing interconnectivity between report text and images and annotations over multiple studies. Unfortunately, most ophthalmology practices cannot create even rudimentary interactive multimedia reports because of incomplete integration and interoperability between imaging modalities, image viewers, and EHRs. Currently only a few manufacturers have imaging devices across multiple imaging technologies (e.g., optical coherence tomography, fundus photography, visual fields). While a vendor’s proprietary viewer systems may support IMR-like display from their modalities, integration for other ophthalmology devices or vendors is not common. The norm being multiple vendors deployed at individual clinic sites, interoperability suffers.

The current field of ophthalmology is overburdened with a high demand of monthly clinic visits with ophthalmologic imaging required to determine if the patient requires treatment for diabetic retinopathy or age-related macular degeneration. Instead of being done in a clinic environment, a more efficient tele-ophthalmology approach includes point-of-care image acquisition at a local pharmacy or optometrist office, or even at home via imaging devices attached to mobile phones [119, 120]. Also as with teledermatology, ophthalmology image data privacy and security are critical, since any images in the glaucoma patient use case above depicting the iris or retina are typically considered PHI [121, 122].

### Current State

Many points shared in the specialties above also commonly apply to ophthalmology. Today, there are no standardized practices on image acquisition, platforms, annotations, or progression analysis of the eye. Standard terminologies for ophthalmologic procedures and anatomy are similarly necessary. There is also variation in how results and images are displayed and stored nationally, and often within different hospitals of the same health system. The clinical workflow of viewing and storing results without standardization creates patient safety and privacy risks and clinical inefficiency. In these unstandardized systems, the images are accessible via printed results, within the device itself, or scanned into the EHR. Clinic notes, again like dermatology, may or may not include pasted images from the encounter. Ophthalmologic structured reporting for imaging is uncommon. Like in pathology, cardiology, and radiology, structured reports created in ophthalmology systems flatten when integrated into the EHR. These barriers prevent IMR growth in ophthalmology.

### Road to the Future

There is a lot to standardize to realize optimal enterprise imaging practices, permit ophthalmology IMR, or integrate ophthalmology data into IMR of other specialties. Ophthalmology providers, information technology societies, and eye care hardware and software manufacturers agreeing on a standard image format and interchange method is an important start. While most new modalities today have the ability to store utilizing DICOM, and DICOM objects are defined for a multitude of different ophthalmology image and measurement types, DICOM penetration into ophthalmology clinics and hospitals is incomplete. For example, the use of PDF and sometimes DICOM Encapsulated PDF for encoding complex information (such as visual fields) is fairly well established, in lieu of using the standard DICOM object that preserves semantically meaningful machine-readable information. In recognition of this pragmatic approach, DICOM has recently been extended to include measurements and other structured data in the metadata header of DICOM Encapsulated PDF objects [123].

Such standards adoption would promote interoperability between sites and permit remote ophthalmology practice access to images captured across the many community optometry locations often seeing patient pathology first. Like radiology and dermatology, having early access to community optometry and ophthalmology images would help triage and track disease over time, detect disease earlier in its course, prevent clinically redundant practices, and provide longitudinal, consistent image data and metadata for IMR.

**Fig. 11:**
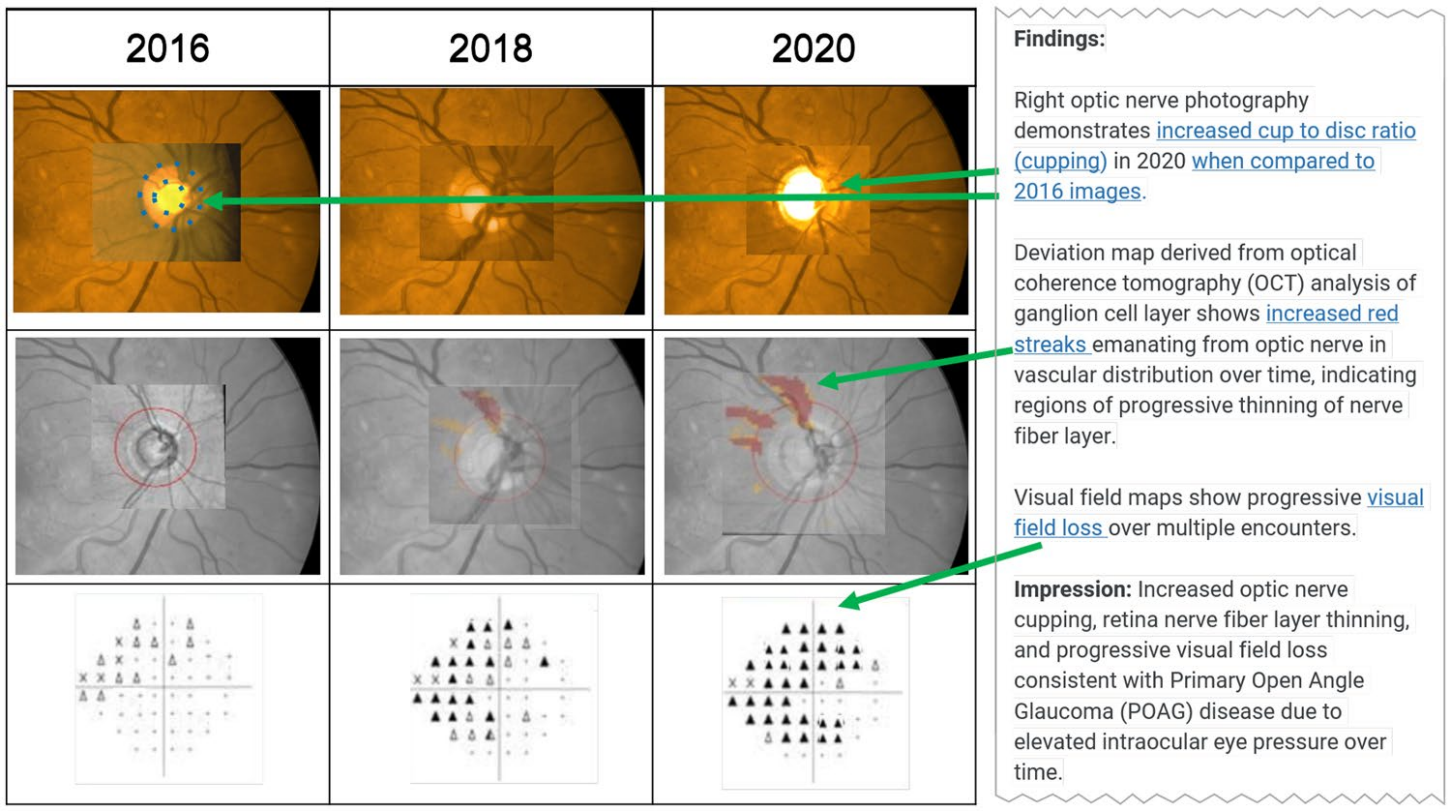
Mockup ophthalmologic interactive multimedia report demonstrating the rate and locations of progression of glaucoma over multiple encounters. The hypertext descriptions in the report connect directly to the image findings, and image modality. (*Images courtesy of D. Luviano MD and Lucas Folio, and have been digitally remastered to mask potential biometric identity*)

Image data and annotation metadata standardized across ophthalmology modality vendors would promote vendor and platform interoperability and offer integration into a universal viewer. In the mockup ophthalmology example below (Fig. 11), the interactive multimedia report contains hyperlinks to images and better reporting results that direct clinicians directly to the imaging results/images without the provider having to search various systems. With use of DICOM and easier integration it is easy to imagine integrating fundoscopic imaging of a retinoblastoma with serial orbital MRI with and without contrast, or a bulging optic disc in intracranial hypertension alongside treatments and CSF pressure measurements from neuroradiology.

## Physical Medicine and Rehabilitation

Physical Medicine and Rehabilitation, also known as PM&R, physiatry, or rehabilitation medicine, works closely with physical therapy, occupational therapy, and preventive medicine. Together with telehealth, these disciplines help enhance and restore functional ability and quality of life to those with physical impairments or disabilities, including, but not limited to, traumatic brain and spinal cord injury, stroke, and musculoskeletal injuries. Rehabilitation medicine aims to maximize individual independence in activities of daily living and return patients to optimal physical performance.

### Current State

**Fig. 12:**
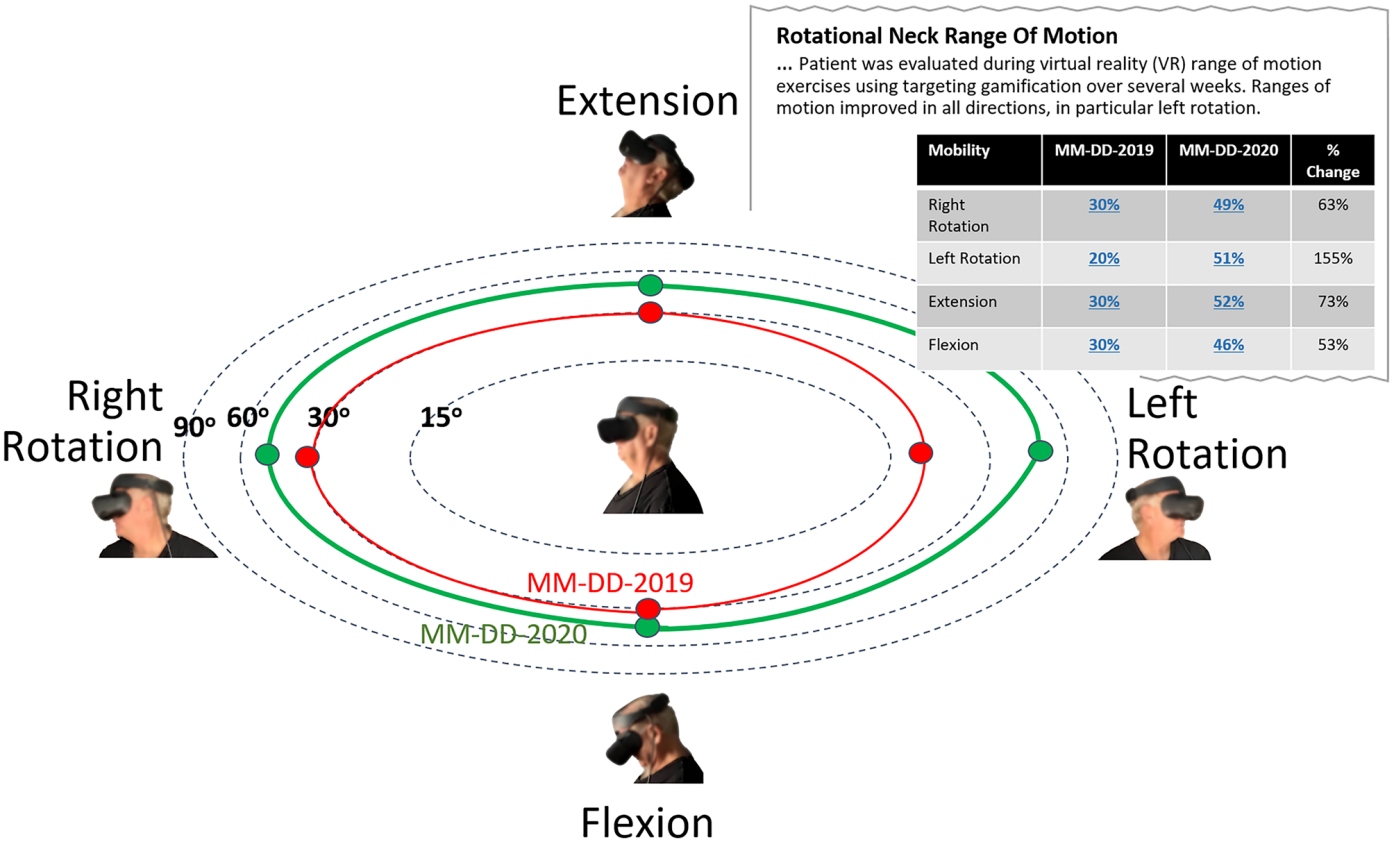
Mock up physiatry interactive multimedia report demonstrating patient tracking a virtual reality insect to gather objective measurements of neck rotational range of motion (ROM) limits in degenerative disease, cervical dystonia, or post trauma patients. The percent rotation values are discretely captured by sensors in the VR goggles and passed to the reporting application. Each current study (green oval) and baseline study (red oval) tabular value is hyperlinked to timestamped external video capturing the point of greatest range of motion in each direction. Dashed ovals represent reference values

Like other specialties, rehab medicine reporting is predominantly plain text. However, telehealth utilization increases have highlighted the need and opportunity for IMR. Rehab medicine image and video over time serve as confirmatory documentation of the patient condition worsening, plateauing, or improving. For example, cervical dystonia, or spasmodic torticollis, can be quantified using cervical range of motion (ROM) to identify involuntary neck muscle contraction (Fig. 12). Before and after still image or video analysis could be performed and incorporated into a graphical timeline to determine the effectiveness of a particular intervention such as chemodenervation with botulinum toxin or stretching therapies. Abnormal gait mechanics can also be quantified virtually using computer software that analyzes a hemiparetic stroke victim or an incomplete spinal cord injury patient working to improve their walking stride [124, 125]. Multimedia reporting in physiatry can also be applied to cerebral palsy patients receiving therapy to improve their gait mechanics. Specifically, Clinical Gait Analysis (CGA) uses sophisticated real-time graphical analysis during treadmill exercises, lending itself to IMR capabilities. Additional graphical reports may include plantar pressures, dynamic kinetic data graphed by cycle and Gait Profile Scores and Movement Analysis Profile [126, 127].

### Road to the Future

Multimedia reporting has a long way to go before becoming commonplace in PM&R. Technical developments supporting more imaging-centric specialties will likely occur before they occur in PM&R. Hopefully, as interoperability improves, PM&R will at least be able to contribute to timeline-based graphical reporting generated by other specialties. For a glioblastoma brain cancer patient, for example, PM&R may contribute function and quality of life baselines and trends, disability management treatments, and symptomatology timestamps to radiology, oncology, or radiation oncology owned IMR.

## Technical Standards

As the HIMSS-SIIM Enterprise Imaging Community IMR Workgroup has discussed in this paper, IMR is generally poorly adopted across the different image-producing specialties. In fact, specialties vary widely in the overall adoption of imaging healthcare IT (HIT) and electronic reporting. For IMR to evolve beyond home-grown and specialized deployments requires HIT technical standard development, integration, implementation, and adoption.

In the past, HIT standards, such as DICOM and HL7, and profiles including those standards, such as IHE’s Scheduled Workflow (SWF), enabled interoperability of image transfer, scheduled patient worklists, and simple report content [128]. The decades-long penetration of these and similar efforts show the value of integrating HIT technical standards. Careful extension or profiling of existing standards or creation of new standards or profiles are similarly needed to address the challenges of advanced interactive reporting. Successful standards development involves not just product vendors and technical experts, but clinicians who can articulate the use cases to be addressed, identify product and standards gaps, and mobilize community adoption.

Although technical standards tend to be designed for a limited number of focused use cases, they are often still moderately flexible, supporting a range of uses. Some of these uses depend on capabilities that the standard declares as optional. Vendors are often reluctant to invest in implementing optional features since there is no guarantee that other systems with which their product must communicate will also implement that optional capability. Even if implemented, systems are often configured to only use baseline capabilities to ensure maximal compatibility. For example, HL7 version 2 report messages (ORUs) support unformatted text content and an HL7-defined simple formatted text content. The messaging standard also supports embedding PDF, RTF, HTML, and other kinds of documents. Even though HL7 v2 supports these advanced features, most software applications utilizing HL7 v2 do not adopt them. Even if the vendor does include the optional, advanced capabilities in a commercial product, hospitals often do not or cannot implement them when even one surrounding application (like PACS or the EHR) in the hospital IT ecosystem cannot interoperate with those features. Even with full technical interoperability between standards and commercial products, end-user physicians may simply choose not to adopt them because they have limited incentives beyond their own specialty. Thus, stitching several standards together into beneficial workflows that can be broadly deployed requires physicians and other users signaling receptiveness to the opportunity, and commercial vendors developing toward meeting those desired workflows. As healthcare systems consolidate and physician reimbursement tilts toward value-based, patient care outcomes-based plans, the HIMSS-SIIM Enterprise Imaging Community believes beneficial cross-specialty technologies like IMR in the EHR will become more appealing.

Organizations like IHE address those gaps through convening expert clinical and technical volunteers to “profile” how the basic and advanced features of multiple standards can be integrated to accomplish cross-standard, innovative use cases beneficial to patient care. IHE has recently proposed profiles for point of care ultrasound and handheld camera encounter-based imaging workflow (EBIW) [129]. With EBIW, the foundational standards already exist for IMR. DICOM Key Object Selection, and Structured Reports, can be used to communicate key images and findings (including finding location in a volume, and lesion tracking between studies). Interactive report content could be encoded in HTML, PDF, RTF, or with additional semantics in HL7 Clinical Document Architecture (XML) or HL7 FHIR DiagnosticReport or Composition [130]. Individual findings can be encoded with HL7 v2 OBX segments in ORU messages, FHIR Observation, or using DICOM Structured Reports. Images and annotations can be retrieved by EHRs, reference viewers, or patient portals via DICOMWeb [131]. Coordination between various systems can leverage HL7 Context Management Specification (CCOW), DICOM UPS (Universal Procedure Step) or the UPS-RS web-based variant, or FHIRcast [132]. The IHE RFD (Retrieve Form for Data Capture), IHE SDC (Structured Data Capture), or its FHIR-based equivalent could be used to assist with the collection of more computable report content [133–135]. Advanced, structured encodings can be leveraged by machine learning and artificial intelligence applications, user-appropriate report rendering, report pre-population, and more efficient contribution to population-based registries.

It is believed that the foundational technical standards for IMR exist; however, we are not yet at the point where a cross-vendor standards-based IMR implementation has been implemented. Interoperability will remain limited unless all systems creating, using, and consuming content agree on terminology for procedure codes, anatomy, feature descriptors, and similar elements. In many cases the need is beyond simple terminology and into ontologies or complete data models and associated search or matching capabilities. Profiles are needed to specify how information flows between modality, PACS, report authoring tool, information systems, and the EHR. These profiles need to document how reports are encoded, expectations of report storage and display systems, how findings and key images are communicated between systems, how the activity context is identified, how users are authenticated, how users create interactive multimedia reports, how users add to existing interactive multimedia reports “owned” by other specialties, and many other aspects. This profiling needs to define how a specific standard or multiple standards can be used to accomplish IMR.

The HIMSS-SIIM Enterprise Imaging Community IMR workgroup has a technical section developing a follow-up paper outlining technical considerations in interactive multimedia report creation, distribution, and consumption. This technical workgroup is scoping a “minimum viable product” IHE Radiology IMR profile for upcoming development.

## Conclusion

The HIMSS-SIIM Enterprise Imaging Community IMR workgroup has outlined the benefits of more visual, interactive reporting of imaging procedures and interventions that output imaging data. These benefits include easy to understand aggregations of multispecialty data, more efficient and engaging data presentation, fewer abstraction or data entry errors, structured and labeled data for further research and downstream quality assurance processes, and likely better collaboration between provider groups. We have defined and demonstrated the current state and future directions of interactive multimedia reporting in several imaging-centric medical specialties through example reports, including images, videos, rich text, tables, graphs, patient event timelines, and hyperlinks. Unfortunately, most specialties today utilize only plain textual descriptions of imaging in dedicated reports or buried in clinical documentation. Multimedia and interactive reports remain uncommon regardless of specialty. Those sites that have adopted IMR subsequently experience limited functionality after that report has transferred into the EHR.

Several critical limitations are preventing widespread adoption of IMR. Heterogeneity in clinical workflows and dataflows in image capture, storage, viewing, and reporting semantics are the first. These exist across specialties and within specialties. Particularly challenging areas include structured data creation by providers to support IMR, EHR integration of native IMR functionality, purchasable applications to aggregate such widespread metadata and multimedia, and having a consistently used imaging format as described above. Interestingly, heavily DICOM-based imaging specialties appear more apt to adopt widespread IMR than do specialties with currently low DICOM penetration. DICOM-based specialties having better capabilities for inherently rich metadata, native support for annotations and key image tagging, image viewing, and image sharing across applications and across physical locations are closer to deep IMR adoption than are those specialties where non-standard, proprietary image formats or consumer image formats without metadata persist.

Incentives may be another reason for limited IMR adoption. With healthcare reimbursement still largely fee-for-service and productivity-based, any activity impairing productivity, even minimally, is opposed. In addition, the personnel and technology needed for the necessary image capture, storage, and curation can be a cost-prohibitive upfront investment in some clinical practices and use cases—dermoscopy in dermatology and whole slide imaging in pathology, for examples—that have no opportunity to offset costs with related charge codes [136–138]. As more healthcare providers and hospitals are financially motivated toward higher quality, higher efficiency care, IMR may become a competitive advantage for practices with interoperable systems that facilitate it, especially if the value of improved communication and patient care can be quantified. As those competitive advantages grow, more practices may grow interested in adopting the workflows, dataflows, and applications supporting IMR. A sub-workgroup emerged from this HIMSS-SIIM Enterprise Imaging Community workgroup focused on the needed IMR technical developments so that industry is prepared as clinicians become more interested in interactive multimedia reporting.
